# Comparative Proteomics Analyses of *Kobresia pygmaea* Adaptation to Environment along an Elevational Gradient on the Central Tibetan Plateau

**DOI:** 10.1371/journal.pone.0098410

**Published:** 2014-06-02

**Authors:** Xiong Li, Yunqiang Yang, Lan Ma, Xudong Sun, Shihai Yang, Xiangxiang Kong, Xiangyang Hu, Yongping Yang

**Affiliations:** 1 Key Laboratory for Plant Biodiversity and Biogeography of East Asia, Kunming Institute of Botany, Chinese Academy of Sciences, Kunming, China; 2 Plant Germplasm and Genomics Center, the Germplasm Bank of Wild Species, Kunming Institute of Botany, Chinese Academy of Sciences, Kunming, China; 3 University of Chinese Academy of Sciences, Beijing, China; 4 Institute of Tibetan Plateau Research, Chinese Academy of Sciences, Beijing, China; Chinese Academy of Sciences, China

## Abstract

Variations in elevation limit the growth and distribution of alpine plants because multiple environmental stresses impact plant growth, including sharp temperature shifts, strong ultraviolet radiation exposure, low oxygen content, etc. Alpine plants have developed special strategies to help survive the harsh environments of high mountains, but the internal mechanisms remain undefined. *Kobresia pygmaea*, the dominant species of alpine meadows, is widely distributed in the Southeastern Tibet Plateau, Tibet Autonomous Region, China. In this study, we mainly used comparative proteomics analyses to investigate the dynamic protein patterns for *K. pygmaea* located at four different elevations (4600, 4800, 4950 and 5100 m). A total of 58 differentially expressed proteins were successfully detected and functionally characterized. The proteins were divided into various functional categories, including material and energy metabolism, protein synthesis and degradation, redox process, defense response, photosynthesis, and protein kinase. Our study confirmed that increasing levels of antioxidant and heat shock proteins and the accumulation of primary metabolites, such as proline and abscisic acid, conferred *K. pygmaea* with tolerance to the alpine environment. In addition, the various methods *K. pygmaea* used to regulate material and energy metabolism played important roles in the development of tolerance to environmental stress. Our results also showed that the way in which *K. pygmaea* mediated stomatal characteristics and photosynthetic pigments constitutes an enhanced adaptation to alpine environmental stress. According to these findings, we concluded that *K. pygmaea* adapted to the high-elevation environment on the Tibetan Plateau by aggressively accumulating abiotic stress-related metabolites and proteins and by the various life events mediated by proteins. Based on the species'lexible physiological and biochemical processes, we surmised that environment change has only a slight impact on *K. pygmaea* except for possible impacts to populations on vulnerable edges of the species' range.

## Introduction

High elevation areas have always attracted the attention of ecologists, especially in light of global climate change. The unique alpine environments affect biological survival and evolution [1] and high-elevation regions are considered to be more sensitive to climate change than other areas [2], [3]. Most alpine plants experience the harsh climates in the high mountain elevations, such as extremely low temperatures, low oxygen concentrations, strong ultraviolet (UV) radiation, aridity and frequently violent wind conditions. Therefore, alpine plants have evolved with many defense mechanisms designed to help them to survive at high elevations. For example, some alpine plants exhibit dwarfism with a small leaf area index, which avoids stress caused by strong wind [4]. A hairy floss covers the leaves and stems of most alpine plants to protect them from low temperatures [5]. Plants can also avoid water loss by deepening root structures and increasing stomatal control [5]. Previous studies demonstrate that alpine plants use several morphological strategies to adapt to the high-elevation environment, but the underlying proteome and physiological mechanisms of the adaptation remain undefined [6]. In addition, physiological and biochemical characteristics are more sensitive to environmental change when compared with more stable morphology characteristics. Therefore, studies in these directions are essential because the impact of climate change on alpine plants may be predictable.

*Kobresia pygmaea* is a dominant species of alpine meadows in the southeastern humid Tibetan highlands and the high alpine pastures of the southern and eastern declivity [7]. Widespread along the alpine elevation gradient, it is found from the lowest slopes (3000 m) to the highest outposts (5960 m) [7], indicating its successful acclimation to the alpine environment. *K. pygmaea* has many excellent features making it viable at many microsites, including resistance to low temperature, drought, trampling, and soil erosion, which are all of great importance in maintaining the stability of the regional environment [8]. Therefore, it is greatly important to understand interactions between *K. pygmaea* and environments. Elevation gradient causes gradient change of environment factors and is considered to be the epitome of horizontal zone. This supplies a good chance for studying alpine plants adaptation to environment conditions. We hypothesized that environmental conditions became more severe with elevation rising; and what physiological and proteomics characteristics *K. pygmaea* applied to survive? In the present study, we selected four elevational gradients to understand the integral adaptation mechanisms of *K. pygmaea*. The results showed that *K. pygmaea* used multiple strategies to acclimate itself to alpine environmental stress; precisely because of this, we hypothesized environment change might have little impact on *K. pygmaea* across a wide area.

## Results

### Vertical investigation and analysis of environmental factors

In our sampling sites (30°30′–30°32′N, 91°03′E), the alpine plant *K*. *pygmaea* is mainly distributed from 4600 to 5200 m in elevation; however, as the elevation reaches 5200 m the populations fall to a very low level. Various factors, including soil temperature, humidity, average sunlight time, average rainfall and average UV radiation exposure, vary along the elevation gradient. Ma et al. [5] found soil temperatures decreased significantly (*P*<0.05) and rainfall increased with increasing elevation at this location during July. Principal component analysis indicated that soil temperature, precipitation (or soil water content) and average UV radiation were three main factors that limited the growth and distribution of *K*. *pygmaea*. Therefore, we mainly considered the effects of temperature, precipitation and solar radiation on *K*. *pygmaea* adaptation along an elevation gradient in our experiments.

### Variation of stomatal density and stomatal aperture length with increasing elevations

**Figure 1:**
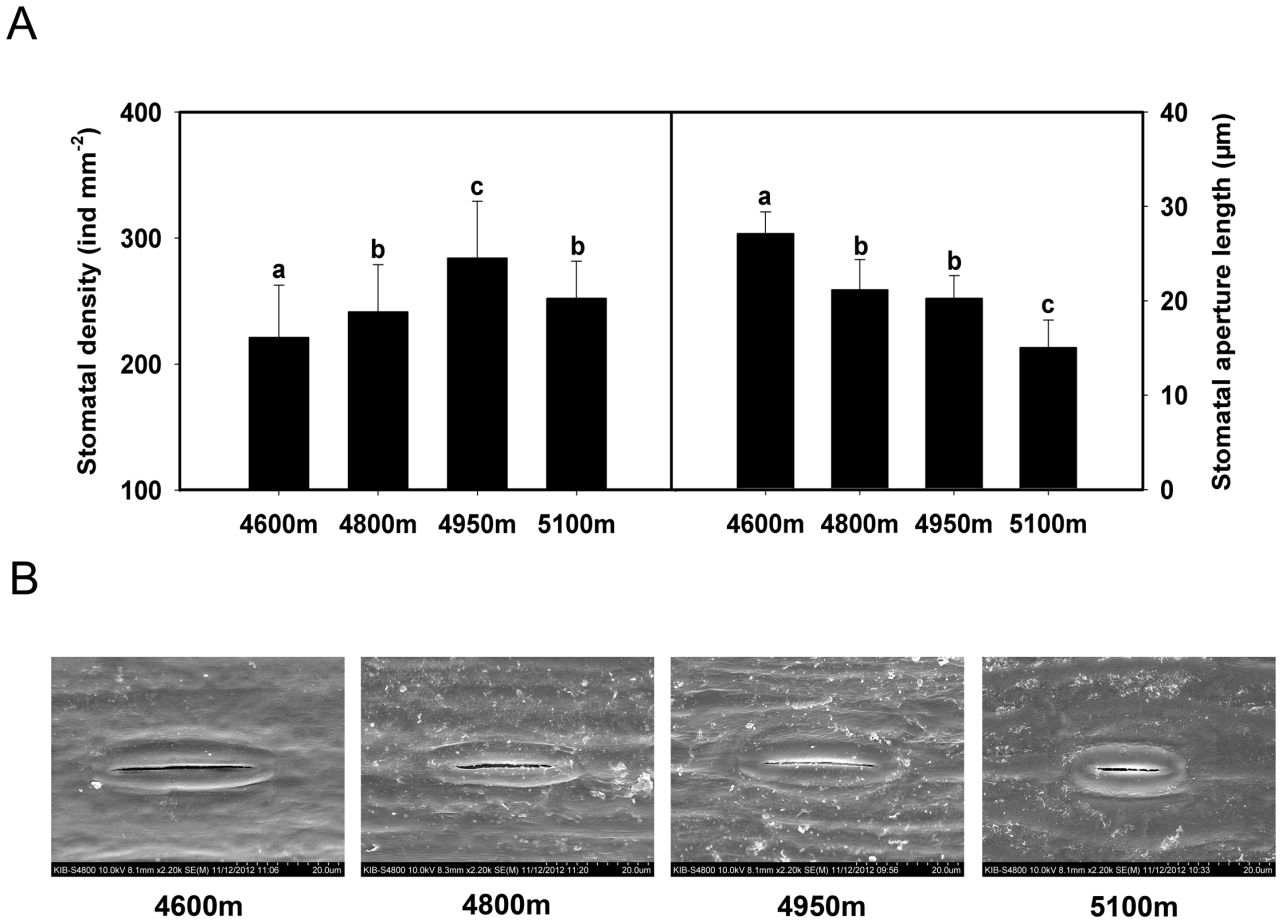
Stomatal characteristics of *K. pygmaea* from different elevations. **A**: Changes in stomatal density (left) and aperture length (right) in *K. pygmaea* along an elevational gradient. **B**: Stomatal shape and size changes of *K. pygmaea* at different elevations at the same magnification. Error bars indicate SE. Means denoted by different letters are significantly different (*P*<0.05) (A).

The stomatal density of *K*. *pygmaea* increases gradually with elevation from 4600 to 4950 m reaching a maximum at 4950 m and then decreasing at 5100 m to a level similar to that at 4800 m (Figure 1A left). In contrast, the stomatal aperture length obviously declined along the elevation gradient, despite little difference observed between 4800 and 4950 m (Figure 1A right & B).

### Differences of photosynthetic pigments content along an elevational gradient

**Figure 2:**
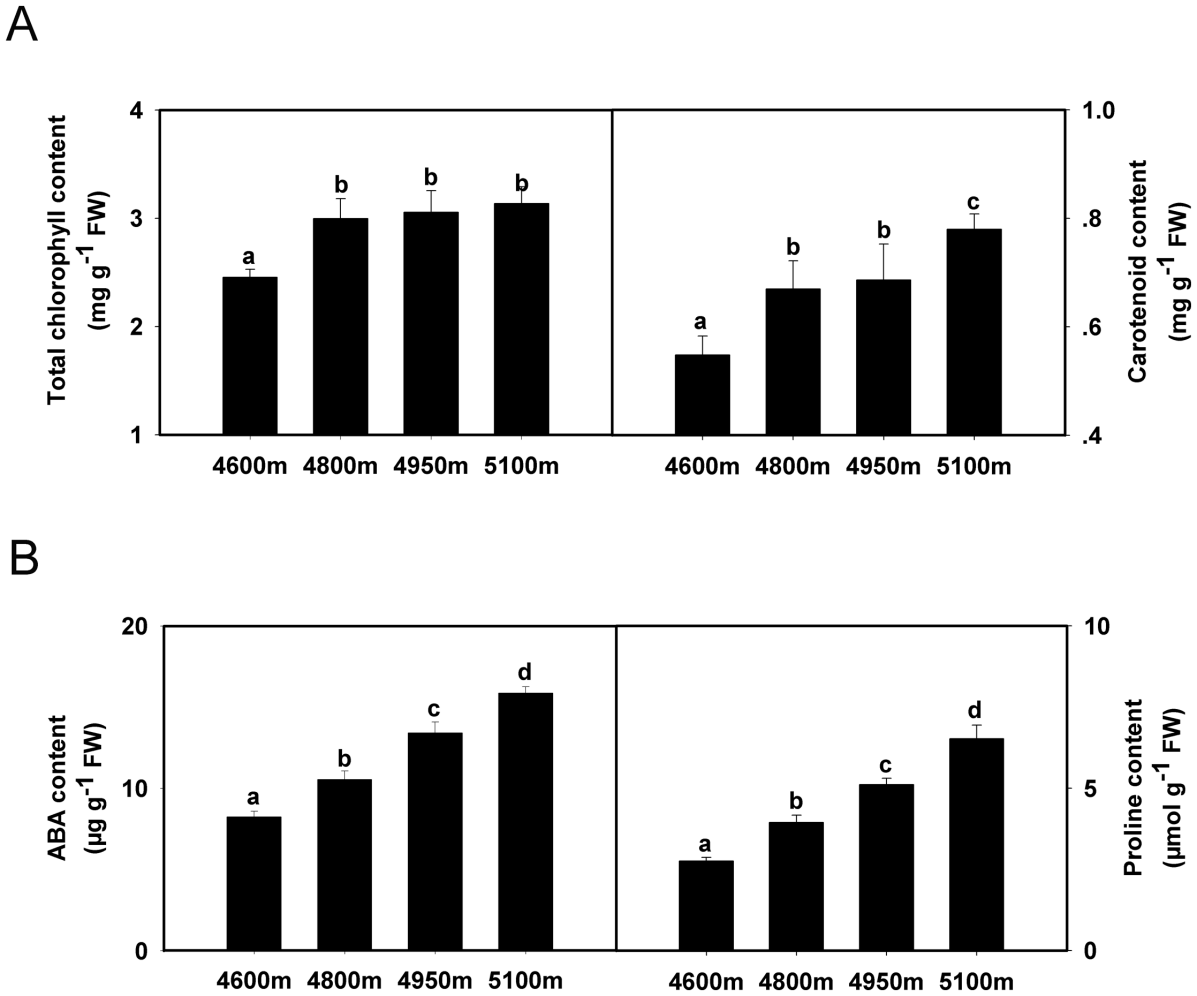
Changes of metabolite content in *K. pygmaea* with elevation increasing. **A**: Changes of photosynthetic pigment content in *K. pygmaea* at different elevations. **B**: Changes of abscisic acid and proline content in *K. pygmaea* along an elevational gradient. Error bars indicate SE. Means denoted by different letters were significantly different (*P*<0.05).

The total chlorophyll content of *K*. *pygmaea* rose significantly from 4600 to 4950 m; it increased only slightly from 4950 to 5100 m (Figure 2A left). Carotenoid content rose continuously with increasing elevation from 4600 to 5100 m (Figure 2A right).

### Changes of proline and abscisic acid content with increasing elevation

The proline and abscisic acid (ABA) content both rose obviously with increasing elevation from 4600 to 5100 m (Figure 2B).

### Dynamics of the antioxidant enzyme system along the elevation gradient

**Figure 3:**
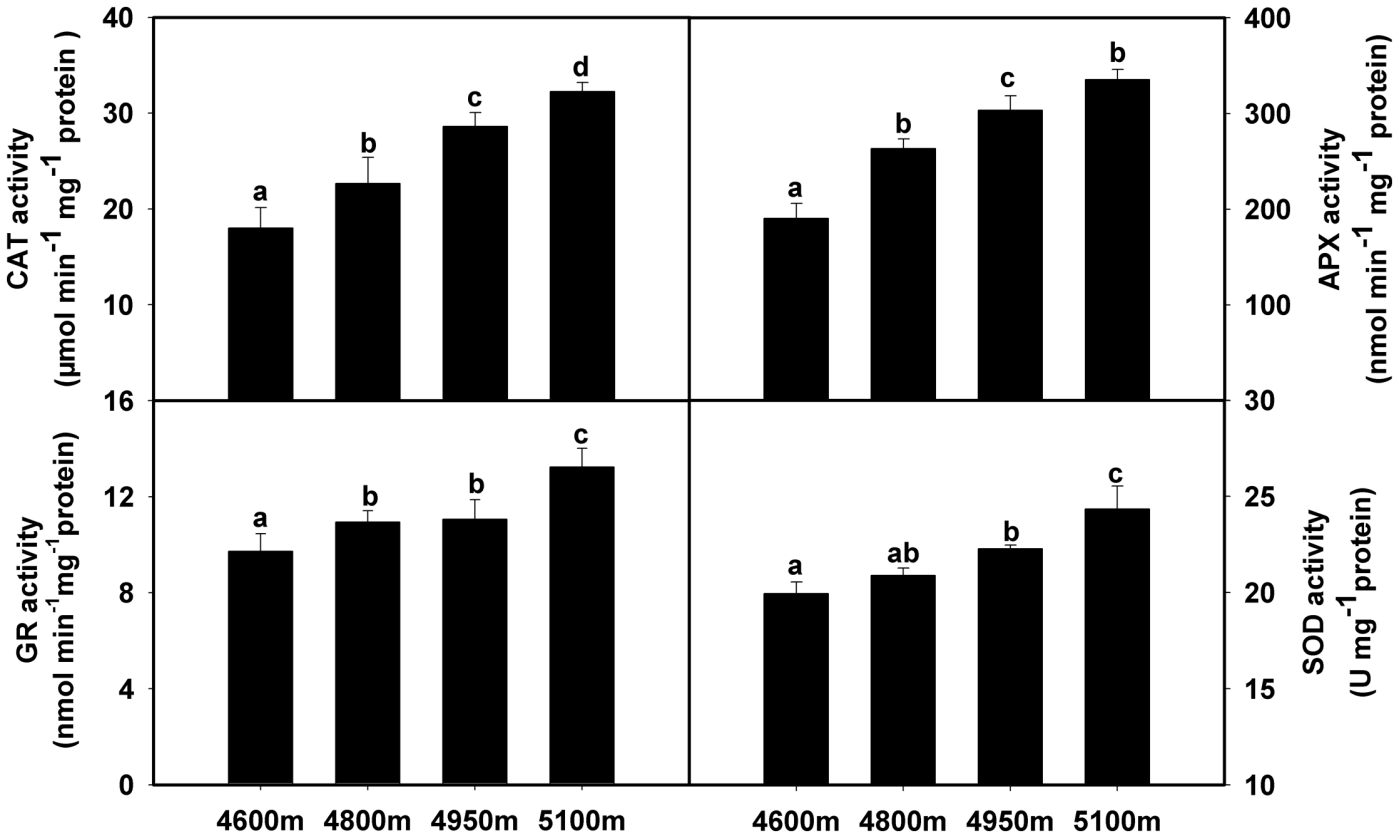
Changes of antioxidant enzyme activities in *K. pygmaea* along an elevational gradient. Error bars indicate SE. Means denoted by different letters were significantly different (*P*<0.05).

To investigate reactive oxygen species (ROS) metabolism of *K. pygmaea* along the elevational gradient, we detected catalase (CAT; EC 1.11.1.6), ascorbate peroxidase (APX; EC 1.11.1.11), glutathione reductase (GR; EC 1.8.1.7), and superoxide dismutase (SOD; EC 1.15.1.1) activities. The results revealed that they exhibited analogous variation. They all increased from 4600 to 5100 m, although the amplitude of change varied at different elevations (Figure 3).

### Protein profiling of *K. pygmaea* adaptation to the alpine environment with increasing elevation

**Figure 4:**
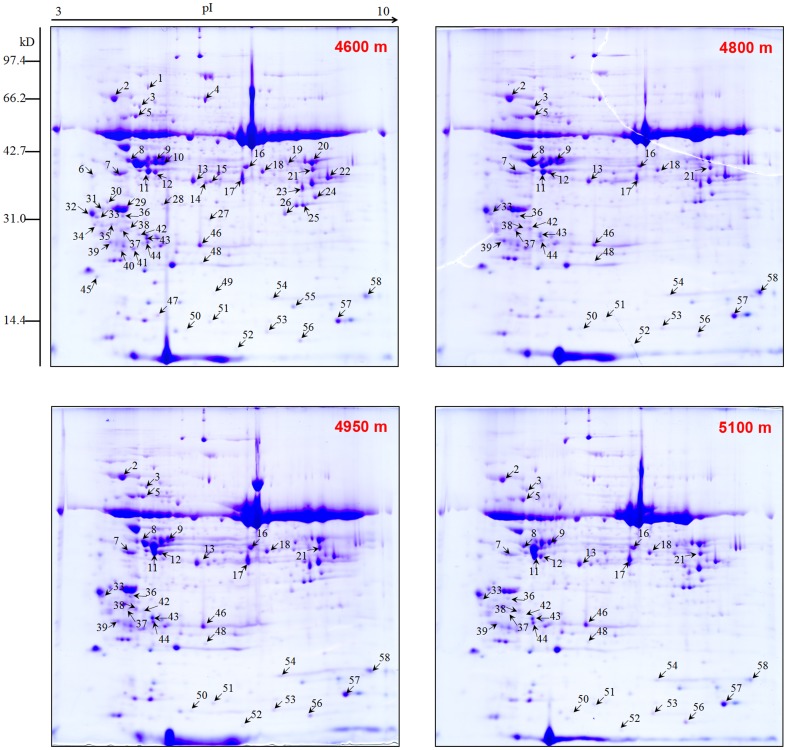
Dynamic protein changes in leaves of *K. pygmaea* along an elevational gradient. All 58 identified protein spots were shown on the 2D gel at 4600

**Figure 5:**
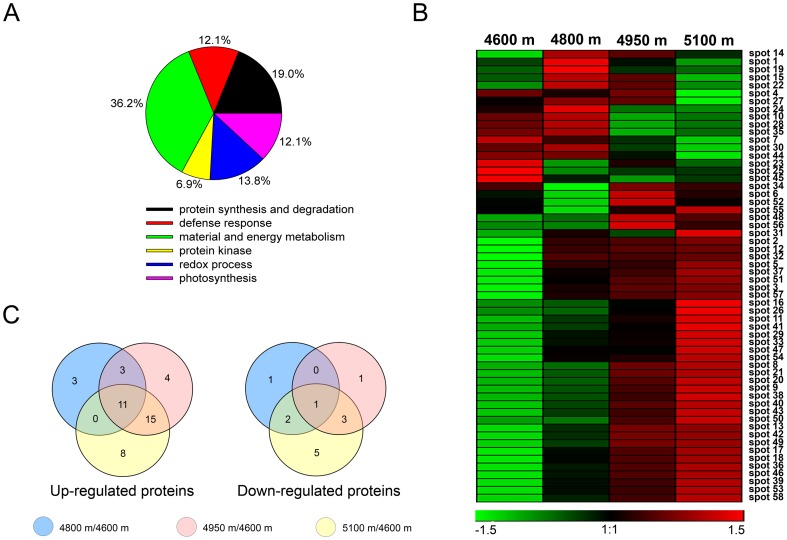
Comparative proteomics analyses results of four samples from different elevations. **A**: Functional classification of the identified proteins based on NCBI annotation. **B**: Hierarchical clustering of the identified protein expression profiles at different elevations. **C**: Venn diagram analysis of differentially expressed proteins at each higher elevations compared with 4600 m. Different colors correspond to the proteins' log-transformed fold-change ratios depicted in the bar at the bottom of the figure (B).

To investigate the response of proteins in *K. pygmaea* to elevational change, we performed two-dimensional electrophoresis (2-DE) to identify the entire protein accumulation profile in *K. pygmaea* from 4600 to 5100 m. We performed three biological replicates and gels were visualized by coomassie brilliant blue (CBB) staining (Figure 4, Figure S1 and S2 in File S1). After staining, proteins were analyzed by PDQuest software (Bio-Rad, Hercules, CA, USA). Under stringent conditions, all differentially displayed proteins were unambiguously identified by MALDI-TOF-MS/MS analysis and compared to the NCBI non-redundant database. 58 protein spots were successfully quantified with significant differential expression changes among the leaf samples from four different elevation locations (4600, 4800, 4950 and 5100 m). All of the proteins demonstrated increased expression (>1.50) or decreased expression (<0.67) at high elevations (4800, 4950 and 5100 m) compared to samples from the lowest elevation (4600 m), which was used as the control (Table 1 and Figure 4). According to NCBI gene annotations, the identified proteins could be classified into six functional groups: material and energy metabolism, protein synthesis and degradation, redox process, photosynthesis, defense response, and protein kinase (Figure 5A and Table 1). A hierarchical cluster analysis was conducted to categorize the proteins that showed differential expression profiles in response to the elevational gradient (Figure 5B). More obviously, antioxidant enzymes, defense response proteins, photosynthesis-related proteins and some material and energy-associated proteins were observed to cluster together, which were up-regulated with increasing elevation (Figure 5B and Table 1). Venn diagram analysis was used to reflect different change patterns of proteins at each higher elevation compared with 4600 m, respectively (Figure 5C), which further proved that many more proteins in *K. pygmaea* were up-regulated rather than down-regulated with an increase in elevation.

**Table 1:** Identification of differentially-expressed proteins in leaves of *Kobresia pygmaea* from different altitudes as analyzed by MALDI-MS/MS.

Spot	Protein name	Acc. No.^a^	Theo. *M*w/p*I* ^b^	Exp. *M*w/p*I* ^c^	SC^d^(%)	Score^e^	Organism	Ratio^f^		
								4800/4600	4950/4600	5100/4600
**Material and energy metabolism**										
4	Transketolase	gi|255541252	78.03/6.76	81.62/6.52	9.66	80	*Ricinus communis*	0.62	1.05	0.13
5	ATP synthase CF1 alpha subunit	gi|197132398	55.0/6.04	58.78/5.15	7.58	300	*Sarcocaulon vanderietiae*	1.38	1.39	1.54
7	Ribulose bisphosphate carboxylase/oxygenase activase, chloroplast precursor	gi|108864712	38.69/5.36	42.09/4.51	19.83	308	*Oryza sativa Japonica* Group	0.78	0.63	0.47
9	Glutamine synthetase 1a	gi|238558241	39.20/5.40	40.465.23	16.43	291	*Lolium perenne*	1.50	2.78	4.59
12	Glutamine synthetase	gi|374676432	39.42/5.82	40.06/5.23	14.33	389	*Cucumis sativus*	2.13	2.30	2.41
13	Glutamine synthetase	gi|374676432	39.42/5.82	41.36/5.80	24.72	396	*Cucumis sativus*	1.43	2.00	2.10
14	Homologous to plastidic aldolases	gi|1781348	38.63/5.89	37.08/5.92	28.01	707	*Solanum tuberosum*	3.43	2.66	1.75
15	Cytosolic malate dehydrogenase	gi|10334493	35.82/5.92	37.52/6.15	29.52	145	*Cicer arietinum*	4.04	2.59	0.62
16	Glyceraldehyde-3-phosphate dehydrogenase B subunit	gi|351726690	48.71/7.1	44.17/7.15	11.09	336	*Glycine max*	1.10	1.30	2.10
17	Glyceraldehyde 3-phosphate dehydrogenase A subunit	gi|166702	37.94/7.00	40.82/7.04	20.00	486	*Arabidopsis thaliana*	1.98	2.61	3.32
18	Fructose-bisphosphate aldolase	gi|40716077	38.64/6.48	42.03/7.47	15.36	259	*Pandanus amaryllifolius*	1.46	1.68	1.92
19	NAD-dependent formate dehydrogenase	gi|4760553	41.45/6.87	42.20/8.03	8.24	65	*Oryza sativa*	2.76	1.15	0.98
21	Fructose-bisphosphate aldolase	gi|226316441	39.17/6.85	42.06/8.36	8.38	104	*Triticum aestivum*	1.17	1.59	1.77
22	NAD dependent epimerase/dehydratase	gi|255542956	42.72/8.52	40.53/8.94	8.92	141	*Ricinus communis*	2.04	1.66	1.01
23	Malate dehydrogenase, glyoxysomal-like	gi|358248482	37.72/8.52	34.81/7.97	26.12	220	*Glycine max*	0.52	0.72	0.57
24	Malate dehydrogenase, glyoxysomal-like	gi|358248482	37.72/8.52	34.58/8.64	26.12	247	*Glycine max*	7.31	0.25	0.19
28	N-glyceraldehyde glyoxysomal-like -2-phosphotransferase-like	gi|8885622	32.00/5.14	35.23/5.32	29.07	145	*Arabidopsis thaliana*	1.07	0.66	0.81
31	Fibrillin	gi|76560800	34.84/5.39	33.45/4.02	6.88	121	*Coffea canephora*	1.18	1.89	1.39
38	Sucrose phosphate phosphatase	gi|46093884	48.36/5.61	25.62/4.74	16.24	183	*Actinidia chinensis*	1.12	1.32	1.56
44	Triose phosphate isomerase cytosolic isoform	gi|192910674	27.48/5.87	25.47/4.97	25.20	131	*Elaeis guineensis*	0.98	0.83	0.63
53	ATP synthase CF1 epsilon subunit	gi|336041850	13.62/5.43	13.12/7.56	51.61	188	*Saccifolium bandeirae*	1.50	1.74	2.18
**Protein synthesis and degradation**										
1	Translation elongation factor EF-G	gi|402753	77.87/5.04	77.18/5.00	7.29	140	*Glycine max*	2.19	1.14	0.79
6	Protein grpE	gi|195637076	36.54/4.53	39.97/3.81	17.38	280	*Zea mays*	0.63	1.28	1.07
10	Elongation factor Tu	gi|357461757	53.26/6.19	43.65/5.49	12.47	274	*Medicago truncatula*	1.07	0.61	0.81
20	Glycine cleavage complex T-protein	gi|17017279	43.92/8.03	44.70/8.71	19.45	131	*Zea mays*	1.18	1.66	2.03
26	50S ribosomal protein L1	gi|226496743	37.24/8.69	36.96/8.12	26.67	99	*Zea mays*	1.27	2.44	10.26
27	Protein disulfide isomerase 1	gi|302851108	57.52/4.85	25.71/6.5	11.17	46	*Volvox carteri f. nagariensis*	2.90	2.15	0.12
30	Hypothetical protein SORBIDRAFT_01g022260	gi|242039737	30.22/5.22	34.10/4.53	19.29	161	*Sorghum bicolor*	1.14	0.74	0.56
32	Nascent polypeptide-associated complex alpha subunit-like protein	gi|226500248	24.34/4.19	30.37/3.79	15.35	98	*Zea mays*	6.00	5.78	6.80
34	Nascent polypeptide associated complex alpha subunit	gi|255569201	14.22/4.24	28.17/3.69	47.33	223	*Ricinus communis*	0.31	1.20	0.94
47	Eukaryotic translation initiation factor 5A-2	gi|350536449	17.71/5.78	15.16/5.23	43.75	233	*Solanum lycopersicum*	1.71	1.67	2.84
55	Peptidyl-prolyl cis-trans isomerase	gi|255547634	27.66/9.58	15.11/8.11	28.91	147	*Ricinus communis*	0.91	1.01	1.09
**Redox process**										
25	Ferredoxin—NADP reductase, putative	gi|255586297	38.65/9.00	34.58/8.64	8.27	162	*Ricinus communis*	0.45	0.54	0.54
40	2-Cys peroxiredoxin	gi|11119229	29.71/5.81	23.77/4.40	15.19	110	*Brassica napus*	1.28	1.75	2.10
41	Oxidoreductase-like (ISS)	gi|308803615	34.21/7.67	24.29/4.66	40.00	79	*Ostreococcus tauri*	1.26	1.45	2.35
42	Ascorbate peroxidase	gi|300837175	27.75/5.83	27.12/4.75	22.00	135	*Citrus limon*	1.36	1.857	1.95
43	Ascorbate peroxidase	gi|24421233	27.76/5.73	26.61/4.98	15.2	52	*Brassica juncea*	1.48	2.11	3.07
46	Manganese superoxide dismutase	gi|1621627	25.26/7.9	22.41/6.20	36.36	112	*Triticum aestivum*	1.31	1.50	1.69
50	Cytosolic superoxide dismutase	gi|169159960	15.48/5.43	12.94/5.91	28.29	131	*Cucumis sativus*	1.06	1.42	1.73
57	Glutathione peroxidase, partial	gi|380862974	17.02/8.4	15.14/8.56	31.13	90	*Dimocarpus longan*	1.75	1.95	2.17
**Photosynthesis**										
36	Chlorophyll a/b binding protein	gi|398599	28.71/5.68	30.63/4.45	18.94	79	*Amaranthus hypochondriacus*	1.36	1.59	1.82
37	Light harvesting chlorophyll a/b-binding protein	gi|3036951	28.56/5.68	28.24/4.32	23.97	96	*Nicotiana sylvestris*	1.38	1.48	1.67
39	Light harvesting chlorophyll a/b-binding protein	gi|3036955	28.48/5.48	25.73/4.19	32.58	89	*Nicotiana sylvestris*	1.25	1.36	1.54
45	Plastid/chloroplast ribosomal protein S10	gi|302851312	19.84/8.76	19.53/4.38	10.73	62	*Volvox carterif. Nagariensis*	0.72	0.61	0.69
49	Chlorophyll a/b binding protein of LHCII type I precursor	gi|2645999	28.12/5.17	25.40/4.21	6.90	231	*Panax ginseng*	1.22	1.57	1.64
54	Photosystem I reaction center subunit IV A	gi|226503797	14.82/9.79	17.27/7.64	16.55	232	*Zea mays*	1.51	1.57	2.08
58	Photosystem I reaction center subunit II	gi|195644572	21.61/9.71	18.20/9.65	40.70	140	*Zea mays*	1.64	2.17	2.98
**Defense response**										
2	Heat shock protein	gi|255570990	75.43/5.35	72.32/4.31	20.77	815	*Ricinus communis*	1.51	1.57	1.67
3	Filamentation temperature-sensitive H 2B	gi|187830110	72.61/5.69	65.74/4.81	31.76	769	*Zea mays*	1.47	1.62	1.70
29	33 kDa manganese stabilizing chloroplast protein	gi|336041494	25.61/5.13	32.43/4.50	28.99	291	*Allium cepa*	1.49	1.59	2.45
33	14-3-3-like protein	gi|37903393	28.98/4.79	31.40/4.05	32.42	324	*Saccharum* hybrid cultivar CP65-357	1.43	1.52	2.24
35	Vf14-3-3c protein	gi|11138320	29.78/4.79	30.27/4.29	20.91	102	*Vicia faba*	1.26	0.28	0.38
48	NAC domain containing protein	gi|108712130	16.20/5.74	18.05/6.13	25.34	107	*Oryza sativa Japonica* Group	1.09	1.71	1.45
51	Heat shock protein hsp20	gi|116783676	38.65/6.16	16.22/6.41	8.41	80	*Picea sitchensis*	1.53	1.77	1.96
**Protein kinase**										
8	Phosphoglycerate kinase, chloroplastic-like	gi|356525742	50.2/7.79	46.00/5.15	7.22	228	*Glycine max*	1.12	1.60	1.79
11	Phosphoribulokinase	gi|226935320	30.59/5.10	25.57/4.87	17.12	150	*Transberingia bursifolia*	1.25	1.43	1.98
52	Nucleoside diphosphate kinase 1-like protein	gi|388564561	16.82/6.3	11.63/6.89	34.23	144	*Saccharum* hybrid cultivar ROC22	0.71	1.42	1.03
56	Nucleoside diphosphate kinase	gi|26245395	16.40/6.91	12.09/8.27	26.85	297	*Glycine max*	1.01	1.84	1.39

**Acc. No.^a^**, database accession numbers according to NCBInr; **Theo.** ***M*** **_w_/p** ***I*** **^b^**, theoretical *M*_w_/p*I*; **Exp.** ***M*** **_w_/p** ***I*** **^c^**, experimental *M*_w_/p*I*; **SC^d^**, sequence coverage; **Score^e^**, mascot search score against the NCBInr database; **Ratio^f^**, different protein spot intensity ratios at different elevations relative to the control (4600 m).

### Changes of malondialdehyde content with increasing elevation

**Figure 6:**
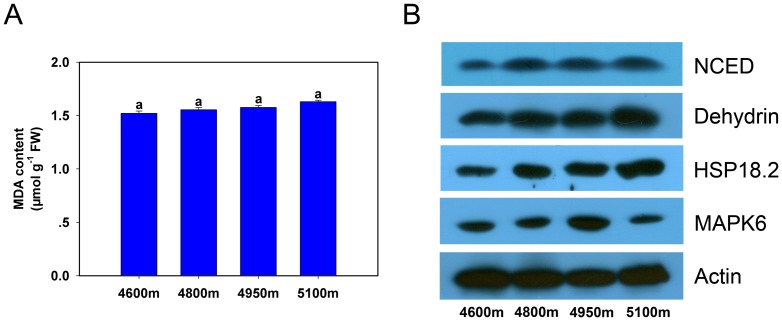
Changes of malondialdehyde content and special proteins expression in *K. pygmaea* with elevation increasing. **A**: Change of malondialdehyde content in *K. pygmaea* along an elevational gradient. **B**: Protein accumulation of 9-cis-epoxycarotenoid dioxygenase, dehydrin, heat shock protein 18.2, and mitogen-activated protein kinase 6 in *K. pygmaea* from different elevations. Actin is included as a protein loading control. Error bars indicate SE. Means denoted by different letters were significantly different (*P*<0.05) (A).

The content of malondialdehyde (MDA) changed slightly and insignificantly from 4600 to 5100 m, (Figure 6A).

### Differences in the expression of specific proteins with elevational change

The results of western blot analysis showed that 9-cis-epoxycarotenoid dioxygenase (NCED), an enzyme regulating ABA synthesis, had continuously increasing expression from 4600 to 5100 m (Figure 6B). Similar variations were found in the expression of dehydrin and heat shock protein 18.2 (HSP18.2) (Figure 6B). The expression of mitogen-activated protein kinase 6 (MAPK6) appeared to be different from these; it increased to a peak from 4600 to 4950 m but fell at the elevation of 5100 m (Figure 6B).

## Discussion

### Coordination between stomatal size and density at high elevations

Stomata allow plants to exchange gases with the surrounding atmosphere. Stomatal density and aperture directly affect the photosynthetic and transpiration rates of plants [9]. Most environment factors can affect the growth and conductance of stomata [10]–[12]; but the relationships between environment factors and stomatal traits are still poorly known because of their multiple correlations in different species [11]–[14]. According to past research [15]–[18], no exact relationship exists between stomatal characteristics and elevational gradients in various species.

In the present study, temperature decreased and moisture increased from 4600 to 5100 m, while stomatal aperture shrank and stomatal density initially rose (4600–4950 m) but then declined (5100 m) (Figure 1A). One reasonable explanation for reduced stomatal aperture is that smaller stomatal sizes are beneficial for *K. pygmaea*, allowing the plant to avoid damage from lower temperature and stronger UV radiation with increasing elevation. However, at the lower altitude, because *K. pygmaea* needs have some degree of drought tolerance due to the lower rates of precipitation, larger stomatal aperture does not help to reduce water loss; thus, *K. pygmaea* remedies this problem by decreasing stomatal density. In addition, environmental conditions may be more unstable at higher elevations, implying a higher frequency of stomatal opening and closing. Because *K. pygmaea* leaves can curl into a needle-shape in the wild, we presume smaller stomatal apparatuses are more flexible in situations where plants have limited leaf areas. That is why the stomatal aperture length significantly decreased at 5100 m (Figure 1A left); nevertheless, to maintain adequate gas exchange space, stomatal density increases, despite a decrease at 5100 m. Speculation may provide some indirect evidence from the accumulation of ABA (Figure 2B left) and its synthase NCED (Figure 6B), since ABA plays an important role in stomatal opening and closing [19], [20]. MAPK signal pathway has also been reported to be involved in the regulation of stomatal development and movement [21], [22]. Interestingly, the expression of MAPK6 was observed to be consistent with variation of stomatal density (Figure 6B), indicating their close relationship.

Most importantly, stomatal development must be affected both positively and negatively by different environmental factors. One result of adaptation to the environment is the coordination between stomatal size and density.

### Photosynthetic pigments and solar radiation

A plant's photosystem is the basis of photosynthesis, and photosynthetic pigments are mainly involved in light absorption and transmission. As elevation increases, solar radiation is enhanced. While this supplies plants with more light, excessive light may damage the photosystem of plants [23]. In our results, two main photosynthetic pigments, chlorophyll and carotenoid, both showed an increasing trend from 4600 to 5100 m (Figure 2A); interestingly, several proteins related to light capture and absorption (spots 36, 37, 39, 54 and 58) displayed similar expression variation (Figure 5B). Chlorophyll content increased significantly from 4600 to 4950 m but only slightly at higher elevations (Figure 2A left). This implies chlorophyll could absorb enough light for photosynthesis; however, excessive light must be prevented to avoid damaging *K. pygmaea*. This may be the reason that chlorophyll content remained steady between 4950 and 5100 m. Carotenoids, as a class of accessory pigments, showed sustained increase from 4600 to 5100 m (Figure 2A right). Apart from the normal function for photosynthesis, carotenoids are believed to help plants absorb excessive light to prevent chlorophyll photo-oxidation by directly assimilating UV radiation in a way that limits damage to plants at high elevations [24], [25].

### The important role of the antioxidant enzyme system in *K. pygmaea*

Reactive oxygen species (ROS) will be generated when plants undergo aerobic metabolism, e.g., photosynthesis and respiration [26]. If superabundant ROS are not removed promptly, plants may experience oxidative stress that may eventually lead cell to death [26]. Thus, plants have evolved scavenging machineries including antioxidant enzymes and antioxidants to keep ROS at physiologically acceptable levels [27].

Here, we determined the activities of four antioxidant enzymes (CAT, APX, SOD and GR) to investigate the response of *K. pygmaea* to the complex environment along an increasing elevational gradient. The results agreed with previous reports that some antioxidant enzyme activities were induced by low temperature, UV radiation or some other types of stresses [28]–[31]. We observed that all four enzyme activities exhibited similar increasing trends (Figure 3), which were consistent with the results related to another alpine herb, *Potentilla saundersiana* [5]. Interestingly, the results were identical for the expression of proteins in the antioxidant system (spots 42, 43, 46, 50 and 57) according to proteomics results (Figure 5B). On the contrary, MDA, which reflected grades of cellular oxidation [32], had little change among the elevational gradient (Figure 6A). This fully certificated that no greater oxidative toxicity to *K. pygmaea* was caused by increasing elevation, implying stimulated antioxidant enzymes played an important role in maintaining ROS homeostasis.

### Effects of material and energy-associated proteins

Plants growing at higher elevations require relatively more energy to respond to serious environmental stress [33]. We observed that the expression of most proteins related to material and energy metabolism changed dramatically at high elevations. Carbohydrates provide the main nutrients used to supply organs with energy. In our experiments, the proteins involved in reducing or non-reducing sugar synthesis were differentially up-regulated with increasing elevation, including glyceraldehyde-3-phosphate dehydrogenase B subunit (spot 16), glyceraldehyde 3-phosphate dehydrogenase A subunit (spot 17), fructose-bisphosphate aldolase (spots 18 and 21), and sucrose phosphate phosphatase (spot 38). These findings agreed with the expression of ATP synthases (spots 5 and 53) (Figure 5B), which regulate ATP synthesis to provide energy for plants. Meanwhile, ribulose bisphosphate carboxylase/oxygenase activase, chloroplast precursor (spot 7), and triose phosphate isomerase cytosolic isoform (spot 44) showed differential down-regulation with increasing elevation (Figure 5B). In addition, other material and energy-associated proteins had irregular variation along the elevational gradient (Figure 5B), suggesting the regulation mechanisms used for carbon metabolism in *K. pygmaea* in response to alpine environmental stress are quite complicated.

Glutamine synthetase (GS) is the key enzyme for plant ammonia assimilation, playing a significant role in normal life activities of plants. Higashi et al. [34] reported that lower GS activity would affect a variety of intracellular enzymes related to nitrogen metabolism and part of the glucose metabolism in radish. In the present study, glutamine synthetase 1a (spot 9) and glutamine synthetase (spots 12 and 13) were up-regulated with increasing elevation (Figure 5B), suggesting the significant regulation of glutamine for this alpine plant in response to environmental stress.

### The roles of abiotic stress-related metabolites and proteins

Both proline and ABA can protect cells against chill and other stressors at various stages of acclimation [35]. Proline helps plants avoid oxidative damage and indicates the presence of a stress response at the cellular level in many plants [36]. It is also believed to mediate osmotic adjustment, stabilize macromolecules, and store carbon and nitrogen for use during stress regimes [37]. ABA is an important plant hormone that modulates responses to abiotic stresses, including cold, heat and drought [38]. In our study, the content of both proline and ABA obviously increased when the elevation rose (Figure 2B). The environmental induction reflected their important roles in responding to low temperature, intense light and UV radiation, etc. with increasing elevation.

ABA synthesis is partly regulated by NCED; and ABA partly regulates the synthesis of dehydrins, which are also induced by cold, salt, and drought stress in plants [39]. Based on our western bolt results, the expression of NCED and dehydrin gradually increased at higher elevations, perfectly matching the change in ABA content (Figure 2B left & 6B). This not only confirmed their close relationships, but also verified the importance of NCED and dehydrin for the adaptation of *K. pygmaea* to the alpine environment.

Heat shock proteins (HSPs) are an important group of protective proteins in eukaryotic organisms, which accumulate when plants are exposed to stressful conditions [40]. HSPs produced in plants can protect an organism's proteins from damage or repair damaged proteins, indicating that the induced formation of heat shock protein will help a plant acquire stress resistance [41]. In this study, both the results of proteomics (spots 2 and 51) and western blot confirmed that rising elevations gradually increased the expression of HSPs in *K. pygmaea* (Figure 6B). This strongly suggested that a high level of HSP expression might confer *K. pygmaea* with greater tolerance to the complex environment of this alpine region.

In addition, other defense response-related proteins, such as filamentation temperature-sensitive H 2B (spot 3), 14-3-3-like protein (spot 33) and NAC domain containing protein (spot 48), generally showed greater expression along the elevational gradient (Figure 5B). The findings supported the idea that the massive accumulation of abiotic stress-related metabolites and proteins improved the ability of *K. pygmaea* to adapt to the alpine environment.

### Effects of the protein synthesis and modification

Proteins are the basis of life and metabolism, so the process of plant defense against harsh environments cannot be separated from the participation of various proteins. In our study, many proteins related to protein synthesis were detected with differential expression patterns along an increasing elevational gradient (Table 1 and Figure 5A). Protein post-translation modifications, like protein phosphorylation and dephosphorylation, can modulate plant response to environmental stress [42]. Phosphoglycerate kinase, chloroplastic-like (spot 8), phosphoribulokinase (spot 11), nucleoside diphosphate kinase 1-like protein (spot 52), and nucleoside diphosphate kinase (spot 56) were differentially regulated with increasing elevation (Figure 5B), which demonstrated the complex regulation mechanisms *K. pygmaea* uses to respond to environmental stress. These findings suggested that protein synthesis and modification were closely related to the interaction between *K. pygmaea* and environment along increasing elevation.

### The predictable effects of environment change on *K. pygmaea* survival and development

Environment is complex and changeable on the Tibetan Plateau, especially when climate change has become an important global issue that needs to be addressed; high-elevation areas are believed to be more sensitive to climate change than other areas [2], [3]. Increasing temperature and changing precipitation patterns are two features of global climate change; they may interactively affect plant growth and development along an elevational gradient.

In this study, temperature and precipitation show different combinations from the lowest to the highest primary range of *K. pygmaea*. Within its range of ecological amplitude, *K. pygmaea* successfully regulates its own physiological and biochemical processes to adapt to the environment, indicating that the species has a certain level of plasticity of adaptation to environmental changes. In another study, investigators analyzed how *K. pygmaea* successfully responded to the diurnal environment at an elevation of 4800 m at the protein level [43]. Taken together, whether on a spatial scale or time scale, *K. pygmaea* plants are able to manipulate the dynamic equilibrium of their internal environment to adapt to environmental change. Therefore, a certain extent of environmental change including climate change will not directly obstruct the survival and development of *K. pygmaea*, since climate change proceeds slowly; however, this idea does not address all issues related to this species. For example, *K. pygmaea* is limited by various environment factors on the fringe of its range, so perceptible environment change may disturb its ability to survive because the species is sensitive and vulnerable on the edge of its range. However, conclusive evidence of the species reaction to changing environment must be gained from long-term field observations or precise simulations of climate change.

## Conclusions

Although *K. pygmaea* is subjected to different environmental conditions at various elevations across its range on the south-facing slope of the Nyainqentanglha Mountains, it is well acclimated to the alpine environment. We found several split-new strategies for *K. pygmaea* to adapt to environmental pressure resulting from the elevational gradient by a comparative proteomics and physiological approach. The strong accumulation of metabolites and proteins involved in abiotic stress and the many life events mediated by proteins were material foundations that help *K. pygmaea* acclimate to the alpine environment. Based on the flexibility of physiological and biochemical processes, we deemed that environment change would have a weak impact on *K. pygmaea* except for those vulnerable populations along the edge of the distribution.

Complex and extensive interactions of various environment factors make it difficult to define the effect on *K. pygmaea* of single factor in the wild. This suggests that carefully controlled laboratory experiments would be necessary to further understand how *K. pygmaea* adapts to the environment. Nevertheless, natural environments are not simple superpositions or offsets of the various factors that can be simulated in the lab; therefore, laboratory experiments cannot represent the actual status of this plant in the wild, even when the impact of each factor on plants is carefully analyzed and controlled. This fully demonstrates that field studies are also crucial to understanding the interaction between *K. pygmaea* and environment along an elevational gradient.

## Materials and Methods

### Ethics Statement

This field study was permitted by the meadow managers, Institute of Geographic Sciences and Natural Resources Research, Chinese Academy of Sciences (CAS), Institute of Tibetan Plateau Research, CAS, and Grassland Station of Damxung County. The work was welcomed by Grassland Station of Damxung County and local herders. There will be no conflict of ethics and interest.

### Experimental samples collection

**Figure 7:**
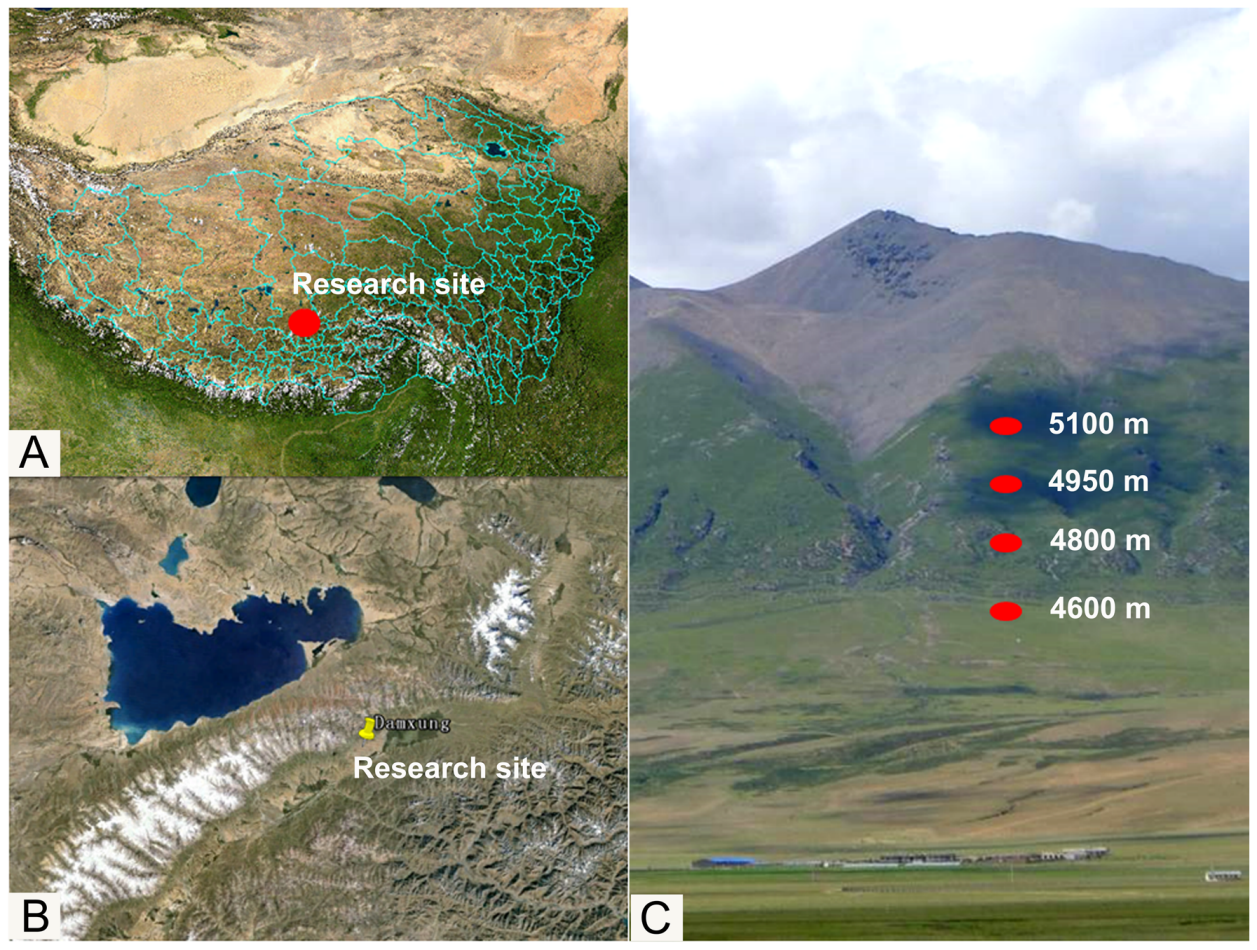
Geographical location of the sampling site. **A**: The research site in the map of the eurasia continent. **B**: The geographical position of the research site on the Tibetan Plateau. **C**: Four sample sites selected at elevations of 4600, 4800, 4950 and 5100 m on the south-facing slope of the Nyainqentanglha Mountains.

This study was conducted in July 2012 on the south-facing slope of the Nyainqentanglha Mountains (30°30′–30°32′N, 91°03′E) near Damxung County, in central Tibet Autonomous Region, China (Figure 7A and B). We selected one general location at an elevation ranging from 4300 to 5600 m where four sample sites were selected at elevations of 4600, 4800, 4950 and 5100 m (Figure 7C). We collected samples at each location at 10:00 a.m. on following sunny days to avoid wide variations in weather and time. Also, we collected samples from within fences built in 2006 to be sure that samples had not been impacted by grazing animals. At each site, 100 to 200 g of healthy leaves from a 10 m^2^ region were randomly selected and immediately frozen by liquid nitrogen for later protein extraction and enzyme analysis. The samples were collected and the experiments were conducted in triplicate.

### Stomatal observation and measurement

Environmental scanning electron microscopy (SEM) was conducted to evaluate the stomatal shape, size, and density using a QUANTA 200 3D (FEI, Tokyo, Japan) equipped with a large-field secondary electron detector and operated at 20 kV. Fresh specimens were fixed in formaldehyde: acetic acid: ethanol (FAA) at 4°C for 24 h. The samples were then dehydrated through a graded ethanol series of 70, 85, 95, and 100% ethanol, each for 45 min. Specimens were critical-point dried using liquid carbon dioxide and then coated with gold and palladium. With the SEM results, five leaves in the same general location were used to record stomatal density and the stomatal aperture length of 20 stomata was measured for each sample.

### Analysis of chlorophyll and carotenoid contents

To measure the chlorophyll content, whole fresh leaves (1 g) were soaked in 10 ml acetone:ethanol (1∶1) for 24 h under darkness. After centrifugation at 12 000×g for 10 min, the supernatant was used for chlorophyll content measurement at absorbance of 645 and 663 nm using a spectrophotometer (Bio-Rad) [44].

### Extraction and determination of malondialdehyde, proline, and abscisic acid content

The malondialdehyde content was determined as described by Duan et al. [45]. Approximately 0.5 g of fresh leaves were homogenized in 10 mL of 10% trichloroacetic acid (TCA) and centrifuged at 12 000×*g* for 10 min. Then, 2 mL of 0.6% thiobarbituric acid in 10% TCA were added to an aliquot of 2 mL of the supernatant. The mixture was heated in a boiling water bath for 30 min and then quickly cooled in an ice bath. After centrifugation at 10,000×*g* for 10 min, the absorbance of the supernatant at 450, 532, and 600 nm was determined. The MDA concentration can be estimated through the formula C (nmol ml^−1^)  = 6.45(A_532_–A_600_) – 0.56A_450_. The MDA concentration was expressed as µmol g^−1^ fresh weight.

The proline content was measured as previously reported [46]. Approximately 0.5 g of fresh leaves were homogenized in 8 mL of 3% aqueous sulphosalicylic acid, and the homogenate was centrifuged at 2 000×*g* for 10 min. Then, 2 mL of the extract were reacted with 2 mL of acidic-ninhydrine and 2 mL of glacial acetic acid for 1 h in boiling water. The reaction mixture was extracted with 4 mL toluene. The chromophore containing toluene was separated and the absorbency read at 520 nm with a spectrophotometer (Bio-Rad).

The abscisic acid quantification was performed using a previously described method [47]. Lyophilized samples from different regions were ground to a fine powder in liquid nitrogen using a mortar and pestle. Triplicate samples (50 mg dry weight each) were extracted with 5 mL of 80% acetone containing 100 mg/mL 2, 6-ditert-butyl-methyl phenol and 500 mg/L citric acid for 16 h at 4°C in the dark. The extracts were further homogenized using a Polytron (Brinkmann Instruments, Westbury, NY, USA) at maximum speed for 1 min and centrifuged at 3000×*g* for 5 min.

### Antioxidant enzyme activity assays

The activities of catalase, ascorbate peroxidase, glutathione reductase, and superoxide dismutase were measured spectrophotometrically by monitoring the change in A240, A290 and A560, respectively [48], [49]. A 5 g leaf sample was homogenized to a fine powder with a mortar and pestle under liquid nitrogen. Then, the powder and pestle were ground with 30 mL of extraction buffer containing 50 mM sodium phosphate buffer (pH 7.0), 0.2 mM EDTA and 2% polyvinylpolypyrrolidone (PVPP) for 10 min. The homogenates were filtered through two layers of cheesecloth and centrifuged at 4°C at 15 000×g for 15 min. Supernatants were desalted on a Sephadex G-50 column (Pharmacia) and this final sample was used to measure the enzymatic antioxidant activities.

### Protein extraction and two-dimensional gel electrophoresis

Protein extraction and two-dimensional electrophoresis were performed according to a previous method [50], with minor medications. Approximately 10 g of leaves from samples collected at elevations of 4600, 4800, 4950, and 5100 m were ground in liquid nitrogen and total soluble proteins were extracted on ice in acetone containing 10% (w/v) TCA and 0.07% (w/v) dithiothreitol (DTT). The homogenates were held at −20°C for 4 h and then centrifuged (8 000×*g*, 30 min, 4°C). The pellets were washed with acetone containing 0.07% (w/v) DTT at −20°C for 30 min and then centrifuged (8 000×*g*, 20 min, 4°C) a total of three times. Finally the pellets were vacuum-dried and then dissolved in lysate (7 M urea, 2 M thiourea, 4% (w/v) CHAPS (3-[(3-cholamidopropyl)-dimethylammonio]-1-propane sulfonate), and 60 mM DTT) for 2 h at room temperature with intermittent shocking, and then the samples were centrifuged (12 000×*g*, 20 min, 20°C). The supernatants were collected for 2-DE experiments with 900 µg of total proteins by a method used previously [51]–[53], which were executed in triplicate.

### In-gel digestion and MALDI-TOF/TOF analysis

Protein spots that showed significant changes in expression with change in elevation were excised manually from colloidal CBB-stained 2-DE gels. Protein digestion with trypsin was first performed; then mass spectrometry analyses were conducted using a MALDI-TOF/TOF mass spectrometer 4800-plus Proteomics Analyzer (Applied Biosystems, Farmington, MA, USA) according to methods previously described [51]–[53].

### Database search

The primary and secondary MS data were transferred into Excel files and used as inputs to search against an NCBI non-redundant database; the search was restricted to viridiplantae (green plants) using the MASCOT search engine (www.matrixscience.com). The search parameters were established as follows: no restriction of protein molecular weight; one missed trypsin cleavage allowed; cysteine treated by iodoacetamide; and oxidation of methionine. The peptide tolerance was 100 ppm and the MS/MS tolerance was 0.25 kD. Protein identifications were validated manually, with at least four peptides matching. The keratin contamination was removed and the MOWSE score threshold was greater than 40 (*P*<0.05). Only significant hits were accepted for the identification of the protein sample based on MASCOT probability analysis.

### Western blotting analysis

SDS-PAGE was performed as described previously [54] using 12% (w/v) polyacrylamide slab gels. For Western blot analysis, the protein samples were electroblotted onto polyvinylidene difluoride (PVDF) membranes using a Trans-Blot cell (Bio-Rad). After transfer, the membranes were probed with the appropriate primary antibodies and HRP-conjugated goat anti-rabbit secondary antibody (Promega, Madison, WI, USA) and the signals were detected using an ECL kit (GE Company, Indianapolis, IN, USA). The primary antibodies (all obtained from Agrisera, Inc., Vannas, Sweden) were diluted as follows: polyclonal antibody against plant 9-cis-epoxycarotenoid dioxygenase (NCED; 1∶3000), dehydrin (1∶3000), heat shock protein 18.2 (HSP18.2; 1∶2000), mitogen-activated protein kinase 6 (MAPK6; 1∶1000), and actin (1∶2000).

### Statistical analysis

Statistical analyses were performed using the statistical Software Package for Social Science (SPSS) version 12.0. One-way ANOVA for all variables was used for testing the treatment differences. Differences were considered significant at the *P<*0.05.

## Supporting Information

File S1 **Supporting Figures.** Figure S1, The second set 2-DE of four *K. pygmaea* samples from different elevations. Figure S2, The third set 2-DE of four *K. pygmaea* samples from different elevations.
(ZIP)
