# Supplementary figures and images for: Comparative Proteomics Analyses of Kobresia pygmaea Adaptation to Environment along an Elevational Gradient on the Central Tibetan Plateau
Source: PLoS One. 2014 Jun 2;9(6):e98410. doi: 10.1371/journal.pone.0098410 (PMC4041879; doi:10.1371/journal.pone.0098410)

## Slide 1
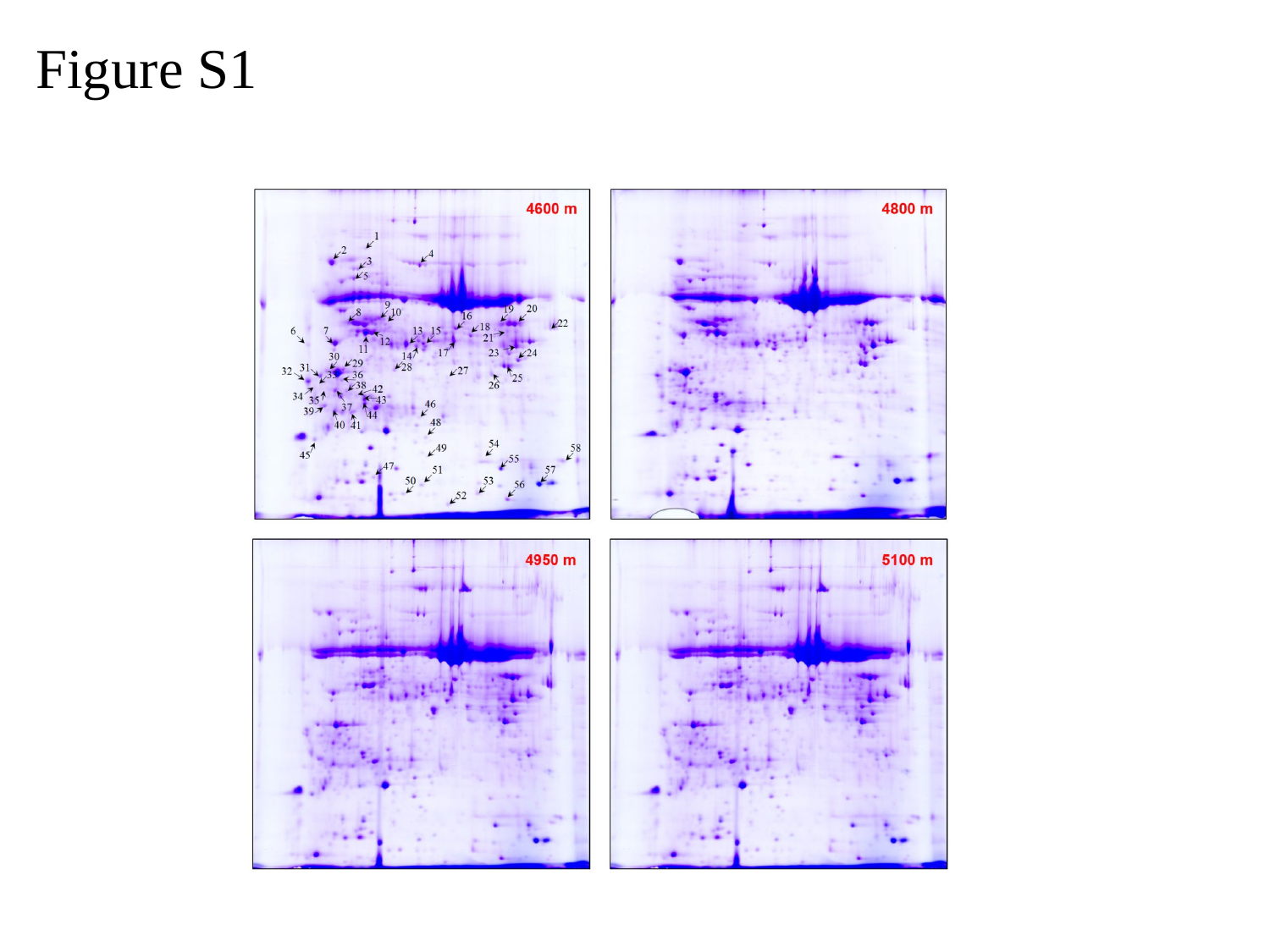

Figure S1

## Slide 2
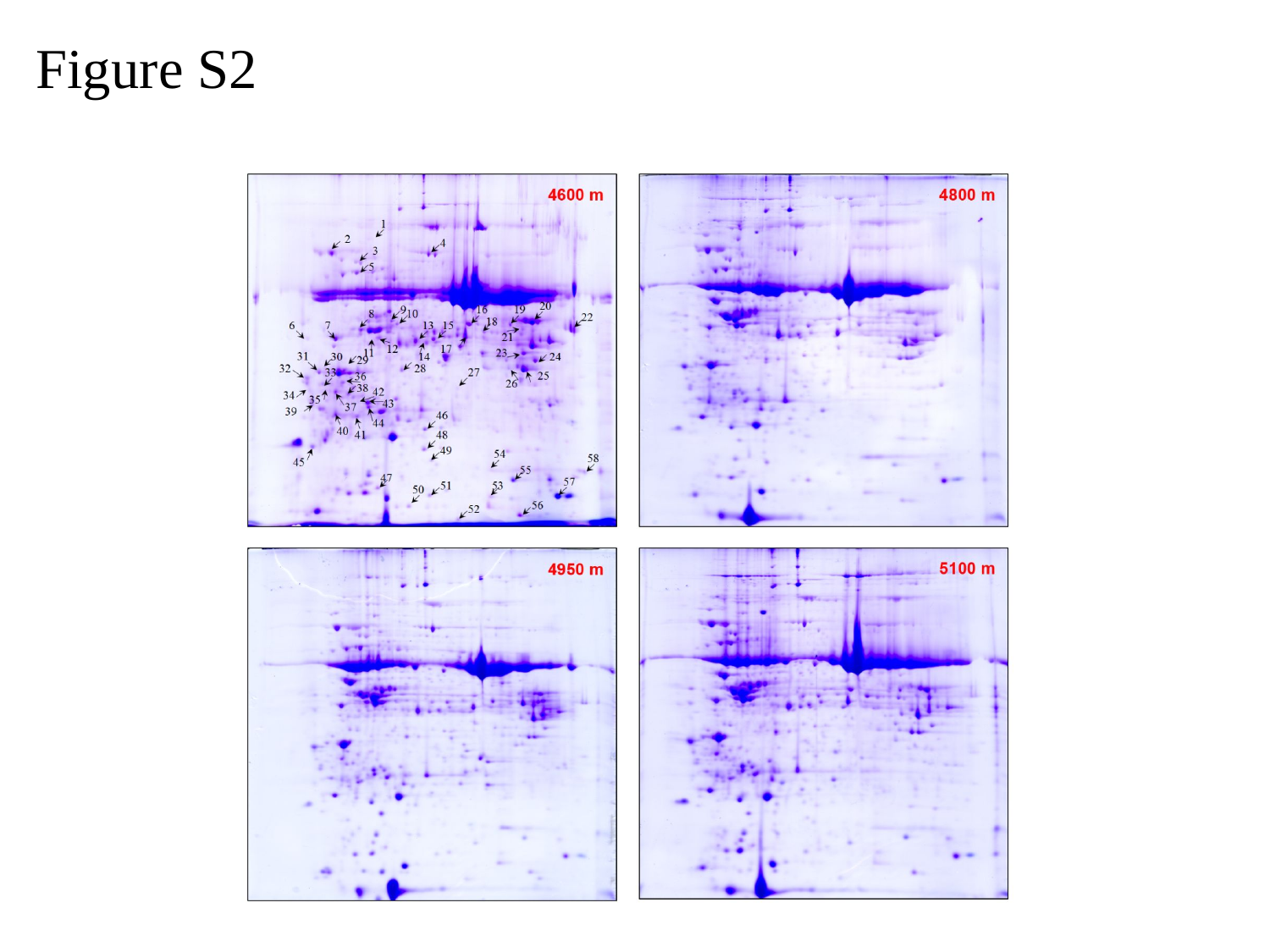

Figure S2

Supplement: File S1 — Supporting Figures. Figure S1, The second set 2-DE of four K. pygmaea samples from different elevations. Figure S2, The third set 2-DE of four K. pygmaea samples from different elevations. (ZIP) [file pone.0098410.s001.zip › Supporting information.pptx]
