# Supplementary material for: Comparative Proteomics Analyses of Kobresia pygmaea Adaptation to Environment along an Elevational Gradient on the Central Tibetan Plateau
Source: PLoS One. 2014 Jun 2;9(6):e98410. doi: 10.1371/journal.pone.0098410 (PMC4041879; doi:10.1371/journal.pone.0098410)
Supplement: File S1 — Supporting Figures. Figure S1, The second set 2-DE of four K. pygmaea samples from different elevations. Figure S2, The third set 2-DE of four K. pygmaea samples from different elevations. (ZIP) [file pone.0098410.s001.zip › Manuscript-2.doc]

**Protein levels analysis of *Kobresia pygmaea* (Cyperaceae) response to diurnal environment on Qinghai-Tibet Plateau**

^1,2,3^Xiong LI ^1,2,3^Yun-Qiang YANG ^1,2,3^Lan MA ^3,4^Shi-Hai YANG ^1,2,3^Xiang-Xiang KONG ^1,2^Xiang-Yang HU^*^ and ^1,2,4^Yong-Ping YANG^*^

^1^(*Key laboratory of biodiversity and biogeography*, *Kunming institute of botany, Chinese Academy of Science*, Kunming, Yunnan 650201, China)

^2^(*Plant Germplasm and Genomics Center, the Germplasm Bank of Wild Species*, *Kunming Institute of Botany, Chinese Academy of Sciences*, Kunming, Yunnan 650201, China)

^3^(*University of Chinese Academy of Sciences*, Beijing, 100049, China)

^4^(*Institute of Tibetan Plateau Research, Chinese Academy of Sciences*, Beijing 100101, China)

***Authors for correspondence.**

Email: [yangyp@mail.kib.ac.cn](mailto:yangyp@mail.kib.ac.cn). Tel: 86-0871-65223234. Fax: 86-0871-83533234.

Email: [huxiangyang@mail.kib.ac.cn](mailto:huxiangyang@mail.kib.ac.cn). Tel: 86-0871-65223069. Fax: 86-0871-835376671.

**Abstract** *Kobresia pygmaea* is an important constructive species of alpine meadow on the Qinghai-Tibet Plateau; its growth and development is tested by both drastic environments of seasonal and inter-annual replacement and diurnal cycle. For a long time, studies about *K. pygmaea* adaption to alpine environment were mainly focused on the long-term adaptation while the diurnal responses were rarely reported. Therefore, we performed comparative proteomics approach, together with antioxidant enzyme assays and western blot to analyze the variations of proteins expression in *K. pygmaea*, with samples collected from south-facing slope of the Nyainqentanglha Mountains on altitude of 4800 m every four hours from 02:00 am to 22:00 pm in a day. The results implicated that *K. pygmaea* was subjected to time-period abiotic environmental stresses, including high temperature, intense light and ultraviolet (UV) radiation in the day and low temperature in the night. To maintain normal life activities, *K. pygmaea* formed a complex set of strategies to deal with the potential damage. These strategies at least contained the plasticity and flexibility of antioxidant system, heat shock proteins (HSPs) accumulation, and abscisic acid (ABA) metabolism. In addition, a potential way named time-special activities was also used to improve efficiency of survival, which meant some important biological processes, such as energy metabolism and photosynthesis, mostly occurred at more suitable time to avoid disadvantageous periods. These results supplied more understandings about alpine plants adaptation to extreme day and night.

**Key words** comparative proteomics, ROS homeostasis, *Kobresia pygmaea*, diurnal environment, Qinghai-Tibet Plateau

**Abbreviations:** 2-DE, two-dimensional gel electrophoresis; ABA, abscisic acid; APX, ascorbate peroxidase; AsA, ascorbic acid; CAT, catalase; CBB, coomassie brilliant blue; CHAPS, 3-[(3-cholamidopropyl)-dimethylammonio]-1-propane sulfonate; DTT, dithiothreitol; EDTA, ethylrnrdiamine tetraacetic acid; GR, glutathione reductase; HSP, heat shock proteins; IEF, isoelectric focusing; MS, mass spectrometer; NCBI, National Center for Biotechnology Information; NCED, 9-cis-epoxycarotenoid dioxygenase; ROS, reactive oxygen species; SDS-PAGE, sodium dodecyl sulfate-polyacrylamide gel electrophoresis; SOD, superoxide dismutase; TCA, trichloroacetic acid; TFA, trifluoroacetic acid; UV, ultraviolet; ZT, zeitgeber time

**Introduction**

The Qinghai-Tibet Plateau, with a mean altitude of more than 4000 m, is considered as roof of the world. Due to the special geographical location and complex terrain, it becomes the sensitive areas and promoter regions of climate change (Wang et al., 2010). With global warming and the ozone weakening, researches about alpine plants response and adaption to environmental factors become more focused hotspots. In addition, the Tibetan Plateau forms typical alpine environment with large temperature change between day and night, intense solar radiation, and some other environmental factors, which are considered to be harsh environment for plants (Zhang et al., 2010). Therefore, alpine plants growing here must have gained special structural characteristics and physiological, ecological adaptation tactics.

Environmental change includes long-term seasonal or inter-annual variation and short-time diurnal turnover. Alpine environments usually show more intense and complex changes. Plants, especially at high altitudes, need to accommodate both long-term and short-time environmental changes to complete normal growth and development. Although the topic of plants adaption to environment has attracted great attentions all the time, studies were almost reflected in the long-term adaptation in the wild (Annicchiarico et al., 1995; Yang et al., 2011; Yang et al., 2012). The situations of plants response to diurnal variation were mainly focused on circadian clock regulation to the diurnal rhythm in model plant, like *Arabidopsis thaliana*, under laboratory conditions (Wang et al., 1998; Somers, 1999; Doyle et al., 2002; Farre et al., 2005; Pruneda-Paz & Kay, 2010; Lai et al., 2012). However, relevant studies were lack of verification in natural field.

Alpine meadow, one widely distributed vegetation type on Tibetan Plateau, is the product of alpine climate. In alpine meadow, *Kobresia pygmaea*, a member of sedge family, often acts as the constructive species. *K. pygmaea* plays a crucial role in maintaining the stability of the regional ecology environment, since it has a strong viability with many excellent features, such as resistance to low temperature, drought, trampling, and soil erosion (Miehe et al., 2008). Therefore, the researches about how *K. pygmaea* adapts to alpine environment and responds to global climate change are very significant. At present, relevant studies were mainly focused on the long-term adaptation primarily from the morphological structure (Yang et al., 2011) and physiological aspects (Yang et al., 2012), but the diurnal response to environmental changes rarely reported. In this study, we adopted comparative proteomics approach, a method widely used for explanation of life phenomenon, together with antioxidant enzyme activity assays and western blot to analyze the variations of proteins expression of *K. pygmaea* in a day. We found the possible roles of antioxidant system, heat shock protein and ABA metabolic in the diurnal environment change for *K. pygmaea*. The results can deepen our understanding of the interaction between alpine plants and natural environment.

**1 Material and methods**

1.1 Samples collection

This study was conducted in July, 2012, on the south-facing slope of the Nyainqentanglha Mountains on altitude of 4800 m (30°31′53″N 91°03′18″E) near Damxung City in central Tibet Plateau. To analyze internal response of *K. pygmaea* to external environment in a day, we collected samples at Zeitgeber time (ZT) 2 (02:00 am), ZT6 (06:00 am), ZT10 (10:00 am), ZT14 (14:00 pm), ZT 18 (18:00 pm) and ZT22 (22:00 pm) respectively. At each time point, 100 to 200 g healthy leaves of *K. pygmaea* from a 25-m^2^ region were randomly selected and immediately frozen by liquid nitrogen for later protein extraction and enzyme assays. The samples were collected and the experiments were conducted in triplicate.

1.2 Detection of environmental factors

The environmental factors including temperature, light intensity, ultraviolet (UV) radiation and atmospheric humidity at different time were observed and measured.

1.3 Antioxidant enzyme assays

Approximately 1 g of leaves from each sample collected at different time were homogenized in extraction buffer (50 mM sodium phosphate pH 7.0, 1 mM EDTA, 1 mM DTT, 1 mM GSH, 1 mM AsA, 5 mM MgCl_2_·6H_2_O, 1% (w/v) PVP-40 and 20% (v/v) glycerin) as the ratio of 100 mg tissue/mL buffer. The homogenates were centrifuged at 12, 000×g for 15 min at 4℃，and the total soluble protein contents in the supernatants were measured according to the Bradford method (Barbosa et al., 2009). The activities of catalase (CAT, EC1.11.1.6), ascorbate peroxidase (APX, EC1.11.1.11), superoxide dismutase (SOD, EC1.15.1.1), and glutathione reductase (GR, EC1.6.4.2) were determined as previously applied method (Beaucham & Fridovic, 1971; Nakano & AsAda, 1981; Varga et al., 2012).

1.4 Protein extraction and two-dimensional gel electrophoresis

Protein extraction and 2D separation was performed according to the methods of Damerval et al. (1986), with some modifications. Approximately 10-20 g leaves from each sample collected at different time were grounded in liquid nitrogen and total soluble proteins were extracted on ice in acetone containing 10% (w/v) trichloroacetic acid (TCA) and 0.07% (w/v) DTT. The homogenates were placed at -20℃ for 4 h and then were centrifuged (8, 000×g, 30 min , 4℃). The resulted pellets were washed with acetone containing 0.07% (w/v) DTT at -20℃ for 30 min and then centrifuged (8, 000×g, 20 min , 4℃), which was repeated for 3 times. The final pellets were vacuum-dried and then dissolved in lysate (7M urea, 2M thiourea, 4%（w/v）CHAPS and 60mM DTT) for 2 h at room temperature with intermittently shocking, and then the samples were centrifuged (12, 000×g, 20 min , 20℃). The supernatants were collected for 2-DE experiments, which were executed in triplicate.

Extracted proteins were first separated by isoelectric focusing (IEF) using gel strips to build an immobilized non-linear pH gradient from 3 to 10 (Immobiline DryStrip, pH 3-10 NL, 17 cm, Bio-Rad, CA, USA) and then by sodium dodecyl sulfate-polyacrylamide gel electrophoresis (SDS-PAGE) using 12.5% polyacrylamides. The strips were rehydrated for 16 h in 320 μl of dehydration buffer containing 900 μg of total proteins and then focused at 20℃ for a total of 64 kV/h with PROTEAN IEF system (Bio-Rad, Hercules. CA). After IEF, the strips were equilibrated for 20 min first in equilibration bufferⅠ(6 M urea, 0.375 M Tris, pH 8.8, 2% (w/v) SDS, 20% (v/v) glycerol and 2% (w/v) DTT) and then in equilibration bufferⅡ(6 M urea, 0.375 M Tris, pH 8.8, 2% (w/v) SDS, 20% (v/v) glycerol and 2% (w/v) iodoacetamide). Following this, the strips were placed over 12.5% (w/v) SDS-PAGE gels for 2-DE, with gel electrophoresis conducted at 25 mA for 5 h. Gels were stained with colloidal Coomassie Brilliant Blue (CBB). After staining, gels were scanned using PDQuest 2D analysis software (Bio-Rad) as Bai et al. (2011) employed, on the basis of their relative volume. To compensate for subtle differences in sample loading or gel staining/destaining during individual experiments, the volume of each spot was normalized.

1.5 Spots digestion and protein identification for Mass Spectrometry analyses

Protein spots displaying significant changes in abundance following plants harvested from different time were excised manually from colloidal CBB stained 2-DE gels using sterile pipette tips. Spots were transferred to 1.5 ml sterile tubes, destained with 50 mM NH_4_HCO_3_ for 1 h at 40℃, reduced with 10 mM DTT in 100 mM NH_4_HCO_3_ for 1 h at 60℃, and incubated with 40 mM iodoacetamide in 100 mM NH_4_HCO_3_ for 30 min. Gels then were minced, air-dried and rehydrated in 12.5 ng/μl sequencing grade modified trypsin (Promega, WI, USA) in 25 mM NH_4_HCO_3_ overnight at 37℃. Tryptic peptides were extracted from the gel grains thrice using 0.1% trifluoroacetic acid (TFA) in 50% acetonitrile. Supernatants were concentrated to approximately 10 μl using a SpeedVac (Savant Instruments Inc., NY, USA) and were desalted using reversed-phase ZipTip pipette tips (C18, P10, Millipore, MA, USA). Peptides were eluted with 50% acetonitrile and 0.1% TFA. Protein spots that fluctuated by more than 1.5-fold and passed the Student’s t-test (*p* < 0.05) were selected and subsequently analyzed by mass spectrometry (MS).

Lyophilized peptide samples were dissolved in 0.1% TFA, and MS analysis was conducted using a 4800 Plus Matrix-assisted Laser Desorption/Ionization-Tandem Time of Flight (MALDI-TOF/TOF) Proteomics Analyzer (Applied Biosystems, MA, USA). MS acquisition and processing parameters were set to reflector-positive mode and an 800–3,500-Da acquisition mass range, respectively. The laser frequency was 50 Hz, and 700 laser points were collected for each sample signal. For secondary MS analysis, 4–6 ion peaks with signal-to-noise ratios exceeding 100 were selected from each sample as precursors. The TOF/TOF signal for each precursor then was accumulated with 2,000 laser pulse. Primary and secondary mass spectra were transferred to Excel files and input in a non-redundant NCBI database (NCBI-nr 20101014) restricted to Viridiplantae (i.e., green plants) using a MASCOT search engine (www.matrixscience.com). The following search parameters were used: no molecular weight restriction, one missed trypsin cleavage allowed, iodoacetamide-treated cysteine, oxidation of methionine, a peptide tolerance of 100 ppm, and an MS/MS tolerance of 0.25 kD. Protein identifications were validated manually with at least 3 peptides matched; the keratin contamination was removed, and the MOWSE threshold was set over 50 (*P<0.05*). According to the MASCOT probability analysis, only significant hits were accepted for the identification of a protein sample.

1.6 Western blot analysis

SDS-PAGE injected with equal amount of total protein was performed as a reference to previous method (Laemmli et al., 1970) using 12% (w/v) polyacrylamide slab gels. Protein samples were electroblotted onto polyinylidene difluoride (PVDF) membranes by using a Trans-Blot cell (Bio-Rad) for western blot analysis. After transfer, the membranes were blocked for 1 h at room temperature. Membranes were probed with the appropriate primary antibodies and HRP-conjugated goat anti-rabbit secondary antibody (Promega, Madison, WI 53711, US), and signals were detected using an ECL kit (GE Company, Evansville, Indiana 47715, US). The primary antibodies were diluted as follows: 9-cis-epoxycarotenoid dioxygenase (NCED) antibody, 1:1000; dehydrin antibody, 1:1000.

**2 Results and discussion**

2.1 Abiotic environment factors incite relational protein expression: proteome analysis

To investigate the response of *K. pygmaea* to diurnal environment change in protein level, we performed 2-DE to identify the whole protein accumulation profile in *K. pygmaea* from ZT2 to ZT22. We performed three biological replicates and gels were visualized by CBB staining (Fig. S1, S2, S3). After staining, proteins were analyzed by PDQuest software (Bio-Rad). Under stringent condition, all differentially displayed proteins were unambiguously identified by MALDI-TOF-MS/MS analysis and searched against the NCBI nonredundant database. In total, comparative proteomics patterns of six different time samples indicated that the expressions of 120 detectable proteins varied by at least 1.5-fold (*P<0.05*) and 60 of these protein spots were positively identified using MALDI-TOF MS (Table 1). According to the NCBI annotations, the identified proteins could be classified into six functional groups: Oxidation reduction processes, stress resistance, energy metabolism, photosynthesis, biosynthesis and metabolism and others (Fig. 2: A). Among these protein spots, we found that the stress resistance, photosynthesis and oxidation reduction processes consisted of most of the identified proteins, occupying 22%, 18% and 18% of all respectively (Fig. 2: A). A hierarchical cluster analysis was conducted to categorize the proteins that showed differential expression profiles at each time (Fig. 2: B).

The study demonstrated that stress resistance proteins response to cold, heat or light and some proteins involved in oxidation reduction processes showed significant changes in expression with diurnal cycle. In particular, a group of important proteins belonging to the antioxidant system, such as ascorbate peroxidase (spot 135) and manganese superoxide dismutase (spot 147) exhibited significant oscillations (Fig. 1, 2: B, C), which exhibited greater expression at ZT6 or ZT14, corresponding to the extreme values of environmental factors.

Heat shock proteins (HSPs), well known in all eukaryotic organisms, play essential roles in various cellular processes when plants are exposed to stressful conditions, such as high or low temperatures, oxygen deprivation, etc. (Siaussat et al., 2013) Under high temperature, HSPs produced in plants can protect organism proteins from damaging or repair damaged proteins, indicating that induced formation of heat shock protein make plant to acquire heat resistance (Kato et al., 1993). In this study, we detected four heat shock-related proteins: putative heat-shock protein (spot 3), heat shock protein, putative (spot 8), stromal 70 kDa heat shock-related protein (spot 9) and heat shock protein hsp20 (spot 173). Three of these proteins showed expression peak at ZT14, and the other one peaked at ZT10 (Fig. 1, 2: B, C). The results fully demonstrated that high temperature in the afternoon (Table S1) was an unfavorable factor for *K. pygmaea*, so large amount of heat shock proteins were induced to play a potential protective effect.

Light is the necessary condition of photosynthesis for plants. In the day light, especially at ZT10, proteins related to light harvesting or light stimulation displayed a higher expression (Fig. 2: B). It may be crucial for the growth of *K. pygmaea* due to volatile environment conditions. For example, in the afternoon, intense light accompanied by high temperature may disturb photosynthesis. Taken together, proteins response to temperature or light, the major abiotic environment factors in diurnal cycle, were incited at special time when they became dominant factor (Table S1).

2.2 Expressions of functional group proteins were aggravated at specific times of the day

Based on the functional classification of comparative proteins, we investigated the expression patterns of group proteins with analogous function. The number of proteins with expressing peak or trough at each time of six functional groups was counted separately (Fig. 3). Indeed, nearly all groups showed the homologous results that proteins expression were elevated at specific times of the day and simultaneously inhibited at some other times. More specifically, proteins involved in stress resistance mainly peaked from ZT2 to ZT14, with significant difference at ZT14 (Fig. 3: A). This illustrated that ZT2 to ZT14 was the adverse period of *K. pygmaea*, especially at ZT14 accompanied by high temperature and intense radiation. Nevertheless, we also found that some resistance proteins reached trough level from ZT18 to ZT2, exhibiting significant difference at ZT2 (Fig. 3: A). These results manifested that some proteins were restrained in the evening or night, while those involved in cold resistance were stated at the same time. Oxidation reduction processes are a set of important courses along with the resilience reaction of plants. Likewise, the expression of proteins related to redox processes reached the maximum from ZT2 to ZT14 and fell to the bottom from ZT18 to ZT2. The difference was that the largest number appeared at ZT6 and ZT18 respectively (Fig. 3: B). Interestingly, we observed analogous appearance on proteins involved in energy metabolism (Fig. 3: C). Collectively, above results suggested that these three processes, resilience reaction, redox process and energy metabolism, were a series of interrelated complex processes in *K. pygmaea*. By doing this, the plants can integrate information between physiological metabolism and environmental change to withstand abiotic challenges.

Apart from above results, energy metabolism, together with biosynthesis and metabolism，are closely contacted with plants photosynthesis. Proteins involved in photosynthesis were more active in the daytime (Fig. 2: B), when the light and temperature was relatively suitable. Precisely, overwhelming majority proteins were animated at ZT10 compared with small amount at ZT14 (Fig. 3: D). This reveled not only that maybe conditions at noon were the most suitable for *K. pygmaea*, but also that photosynthesis was under suppression in the afternoon. Previous studies have certificated that strong light (Poulson et al., 2006), high temperature (Zhou et al., 2010; Yu et al., 2013) and UV radiation (Lud et al., 2002) obstruct photosynthesis through complex mechanism; nevertheless, plants also have evolved a set of defense mechanisms (Liu et al., 2012). Low temperature is regarded as an important interference factor for many physiological processes, and photosynthesis is one of the most obvious processes affected by low temperature (Oquist et al., 1993; Liu et al., 2012). This is why many proteins with various functions exhibited expression trough at midnight (Fig. 3). As photosynthesis is essential for plants, in natural environment, the strategic that efficient photosynthesis concentrated in a short time is highly beneficial to *K. pygmaea* in the long term. Extreme expression of proteins involved in biosynthesis and metabolism (Fig. 3: E) and some other functions (Fig. 3: F) were observed in each time without significant difference, indicating participation throughout the whole day. In summary, expressions of functional group proteins were exacerbated at appropriate times of the day in this research. The temporal allocation may be favorable for *K. pygmaea* to maintain normal physiological activities in the complicated and changeable environment.

2.3 Antioxidant enzyme activities exhibited changes in volatility

Plants will produce reactive oxygen species (ROS) when undergone aerobic metabolism, e.g., photosynthesis and respiration (Apel & Hirt, 2004). If the generation of ROS is not removed timely, plants may experience oxidative stress due to breaking the homeostasis in cellular redox state that may eventually lead to cell death (Apel & Hirt, 2004). Thus, plants have evolved scavenging machineries with antioxidant enzymes and antioxidants to keep ROS at physiologically permissive levels (Mittler, 2002).

Here we determined the activities of four antioxidant enzymes (CAT, APX, SOD, and GR) to investigate the response of *K. pygmaea* to the complex external environment. We observed that all four enzyme activities exhibited similarly fluctuation in the day (Fig. 4), which peaked from ZT2 to ZT6 or from ZT14 to ZT18 and dipped at ZT10 or ZT22. Interestingly, the results were identical to proteomics results of antioxidant system observed above. Our results are consistent with previous reports that some antioxidant enzyme activities, such as APX, GR, and SOD increased under low temperature or heat stress (Lee & Lee, 2000; Lou et al., 2011; Liu et al., 2012; Yu et al., 2013). Thus, the results suggested unfriendly abiotic environment e.g., cold in the mid-night, heat and UV radiation in the afternoon, could induce the production of ROS in *K. pygmaea*. To prevent the potential threat, plants can improve corresponding antioxidant enzyme activities to keep ROS homeostasis.

Previous study reported that circadian clock regulated ROS production and scavenging in *Arabidopsis thaliana* (Lai et al., 2012), and a core feedback loop of circadian clock has been found in plants (Wang & Tobin, 1998; Alabadi et al., 2001; Pruneda-Paz & Kay, 2010). We hypothesized that antioxidant enzyme activities of *K. pygmaea* also would be regulated by circadian clock, despite lack of appropriate mechanism. Nevertheless, plants in natural field, especially on Qinghai-Tibet Plateau, may suffer sudden stress from unstable environment factors, resulting in irregular fluctuation of ROS production. In the present study, we confirmed the truth of that *K. pygmaea* coordinated ROS metabolic processes with the external environment to avoid damaging the plants.

2.4 Abscisic acid and dehydrin might participate in a variety of physiological regulation

To detect some substances played important roles in plant life activities from the perspective of protein, we performed immunoblot analysis with specific antibodies against abscisic acid (ABA) synthase and dehydrin. ABA is an important plant hormone modulating seed dormancy, germination, stoma closure and responses to abiotic stresses, including low temperatures, drought and high temperature (Fujita et al., 2006). Its synthesis is mediated by ABA synthases, like 9-cis-epoxycarotenoid dioxygenase (NCED) in plants. Dehydrins, belonging to a multi-family of proteins, are present in plants and induced by cold, salt, ABA and drought stress (Kosova et al., 2011). In the results of western blot, we found NCED was highly accumulated at ZT2, ZT6, ZT14, ZT18 and ZT22, but with little expression at ZT10 (Fig. 5), implying similar regularity about production of ABA. Combined with environmental change, the result signified ABA was involved in *K. pygmaea* resistance to cold, heat and UV radiation. In spite of lower expression at ZT14 compared with ZT2, ABA was also induced in the afternoon by heat to regulate stomas closure, may be this partly explained many proteins related to photosynthesis were down-regulated. As for dehydrins, we observed the same expression variation (Fig. 5). This suggested dehydrins were partly induced by ABA and played important roles in response to extreme conditions. Overall, we found the ABA synthase and dehydrin were altered with external conditions, which insinuated they were involved in a variety of physiological regulation. Furthermore, the fact that both ABA synthase and dehydrin displayed minimal expression at ZT10 indicated this time might be the more appropriate condition for *K. pygmaea* as regarded above once again.

**3 Conclusions**

In the present study, we investigated and discussed the mode of *K. pygmaea* response to the natural environment during day and night on the protein level. Several categories of proteins were particularly noticed to show apparent volatility changes with the ups and downs of environmental factors, which exhibited strong plasticity and flexibility. By analyzing the intrinsic link, we found that *K. pygmaea* might suffer from time period abiotic environmental stresses, including high temperature, intense light and UV radiation in the daytime and low temperature in the night during a diurnal cycle. To maintain normal life activities, *K. pygmaea* would not stand still and formed a complex set of strategies to fight against the potential damage. These strategies at least contained antioxidant system, HSPs accumulation, ABA metabolism. In addition, a lot of important activities, such as energy metabolism and photosynthesis, mostly occurred at more suitable time to avoid disadvantageous periods, which were designated as time-special activities. Briefly, the plasticity and flexibility of some functional proteins was the material basis of *K. pygmaea* adaptation to the large diurnal environmental changes. These results will improve our understanding of the interaction between alpine plants and natural environment.

**Competing interests**

The authors declare that they have no competing interests.

**Authors’ contributions**

XL drafted the manuscript and participated in the data collection, sample preparation and proteomic analysis. YY participated in the data collection and sample preparation. LM participated into the data collection and sample preparation. SY and XK help to collect the sample. XH designed the experiments and drafted the manuscript. YY conceived of the study, and participated in its design and coordination and helped to draft the manuscript. All authors read and approved the final manuscript.

**Acknowledgements**

This work was supported by the Young Academic and Technical Leader Raising Foundation of Yunnan Province (No.2012HB041); the National Natural Sciences Foundation of China (No. 31170256) and the Major State Basic Research Development Program (2010CB951700).

**References**

Alabadi D, Oyama T, Yanovsky MJ, Harmon FG, Mas P, Kay SA. 2001. Reciprocal regulation between TOC1 and LHY/CCA1 within the Arabidopsis diurnal clock. Science 293: 880-883.

Annicchiarico P, Bozzo F, Parente G, Gusmeroli F, Mair V, Marguerettaz O, Orlandi D. 1995. Analysis of adaptation of grass/legume mixtures to Italian alpine and subalpine zones through an additive main effects and multiplicative interaction model. Grass and Forage Science 50: 405-413.

Apel K, Hirt H. 2004. Reactive oxygen species: Metabolism, oxidative stress, and signal transduction. Annual Review of Plant Biology 55: 373-399.

Bai X, Yang L, Yang Y, Ahmad P, Yang Y, Hu X. 2011. Deciphering the protective role of nitric oxide against salt stress at the physiological and proteomic levels in maize. Journal of Proteome Research 10: 4349-4364.

Barbosa H, Slater NKH, Marcos JC. 2009. Protein quantification in the presence of poly(ethylene glycol) and dextran using the Bradford method. Analytical Biochemistry 395: 108-110.

Beaucham.C, Fridovic.I. 1971. Superoxide dismutase - improved assays and an assay applicable to acrylamide gels. Analytical Biochemistry 44: 276-&.

Damerval C, Devienne D, Zivy M, Thiellement H. 1986. Technical improvements in two-dimensional electrophoresis increase the level of genetic-variation detected in wheat-seedling proteins. Electrophoresis 7: 52-54.

Doyle MR, Davis SJ, Bastow RM, McWatters HG, Kozma-Bognar L, Nagy F, Millar AJ, Amasino RM. 2002. The ELF4 gene controls diurnal rhythms and flowering time in *Arabidopsis thaliana*. Nature 419: 74-77.

Farre EM, Harmer SL, Harmon FG, Yanovsky MJ, Kay SA. 2005. Overlapping and distinct roles of PRR7 and PRR9 in the Arabidopsis diurnal clock. Current Biology 15: 47-54.

Fujita M, Fujita Y, Noutoshi Y, Takahashi F, Narusaka Y, Yamaguchi-Shinozaki K, Shinozaki K. 2006. Crosstalk between abiotic and biotic stress responses: a current view from the points of convergence in the stress signaling networks. Current Opinion in Plant Biology 9: 436-442.

Kato S, Yamagishi K, Tatsuzawa F, Suzuki K, Takano S, Eguchi M, Hasegawa T. 1993. Identification of cytoplasmic and nuclear low-molecular-weight heat-shock proteins in tomato fruit. Plant and Cell Physiology 34: 367-370.

Kosova K, Vitamvas P, Prasil IT. 2011. Expression of dehydrins in wheat and barley under different temperatures. Plant Science 180: 46-52.

Laemmli UK, Beguin F, Gujerkel.G. 1970. A factor preventing major head protein of bacteriophage T4 from random aggregation. Journal of Molecular Biology 47: 69-&.

Lai AG, Doherty CJ, Mueller-Roeber B, Kay SA, Schippers JH, Dijkwel PP. 2012. DIURNAL CLOCK-ASSOCIATED 1 regulates ROS homeostasis and oxidative stress responses. Proceedings of the National Academy of Sciences of the United States of America 109: 17129-17134.

Lee DH, Lee CB. 2000. Chilling stress-induced changes of antioxidant enzymes in the leaves of cucumber: in gel enzyme activity assays. Plant Science 159: 75-85.

Liu YF, Qi MF, Li TL. 2012. Photosynthesis, photoinhibition, and antioxidant system in tomato leaves stressed by low night temperature and their subsequent recovery. Plant Science 196: 8-17.

Lou B, Xu DD, Xu HX, Zhan W, Mao GM, Shi HL. 2011. Effect of high water temperature on growth, survival and antioxidant enzyme activities in the Japanese flounder Paralichthys olivaceus. African Journal of Agricultural Research 6: 2875-2882.

Lud D, Moerdijk TCW, Van de Poll WH, Buma AGJ, Huiskes AHL. 2002. DNA damage and photosynthesis in Antarctic and Arctic *Sanionia uncinata* (Hedw.) Loeske under ambient and enhanced levels of UV-B radiation. Plant Cell and Environment 25: 1579-1589.

Miehe G, Mlehe S, Kaiser K, Liu JQ, Zhao XQ. 2008. Status and dynamics of *Kobresia pygmaea* ecosystem on the Tibetan plateau. Ambio 37: 272-279.

Mittler R. 2002. Oxidative stress, antioxidants and stress tolerance. Trends in Plant Science 7: 405-410.

Nakano Y, AsAda K. 1981. Hydrogen-peroxide is scavenged by ascorbate-specific peroxidase in spinach-chloroplasts. Plant and Cell Physiology 22: 867-880.

Oquist G, Hurry VM, Huner NPA. 1993. Low-Temperature Effects on Photosynthesis and correlation with freezing tolerance in spring and winter cultivars of wheat and rye. Plant Physiology 101: 245-250.

Poulson ME, Boeger MRT, Donahue RA. 2006. Response of photosynthesis to high light and drought for *Arabidopsis thaliana* grown under a UV-B enhanced light regime. Photosynthesis Research 90: 79-90.

Pruneda-Paz JL, Kay SA: An expanding universe of diurnal networks in higher plants. *Trends in* Plant Science 2010, 15:259-265.

Siaussat D, Laparie M, Maria A, Renault D. 2013. Heat shock protein responses to salinity, food deprivation, and temperature in the invasive ground beetle *Merizodus soledadinus* at the Kerguelen Islands. Polar Biology 36: 201-209.

Somers DE. 1999. The physiology and molecular bases of the plant diurnal clock. Plant Physiology 121: 9-19.

Varga B, Janda T, Laszlo E, Veisz O. 2012. Influence of abiotic stresses on the antioxidant enzyme activity of cereals. Acta Physiologiae Plantarum 34: 849-858.

Wang Y, Meng LL, Yang YP, Duan YW. 2010. Change in floral orientation in *Anisodus luridus* (Solanaceae) protects pollen grains and facilitates development of fertilized ovules. American Journal of Botany 97: 1618-1624.

Wang ZY, Tobin EM. 1998. Constitutive expression of the DIURNAL CLOCK ASSOCIATED 1 (CCA1) gene disrupts diurnal rhythms and suppresses its own expression. Cell 93: 1207-1217.

Yang Y, Wang GX, Klanderud K, Yang LD. 2011. Responses in leaf functional traits and resource allocation of a dominant alpine sedge (*Kobresia pygmaea*) to climate warming in the Qinghai-Tibetan Plateau permafrost region. Plant and Soil 349: 377-387.

Yang Y, Wang GX, Yang LD, Guo JY, Li N. 2012. Physiological responses of *Kobresia pygmaea* to warming in Qinghai-Tibetan Plateau permafrost region. Acta Oecologica-International Journal of Ecology 39: 109-116.

Yu C, Huang SJ, Hu XM, Deng W, Xiong C, Ye CH, Li Y, Peng B. 2013. Changes in photosynthesis, chlorophyll fluorescence, and antioxidant enzymes of mulberry (*Morus ssp*.) in response to salinity and high-temperature stress. Biologia 68: 404-413.

Zhang LY, Turkington R, Tang Y. 2010. Flowering and Fruiting Phenology of 24 Plant Species on the North Slope of Mt. Qomolangma (Mt. Everest). Journal of Mountain Science 7: 45-54.

Zhou HH, Chen YN, Li WH, Chen YP. 2010. Photosynthesis of *Populus euphratica* in relation to groundwater depths and high temperature in arid environment, northwest China. Photosynthetica 48: 257-268.

**Figure legends**

**Fig. 1**: Representative BR-20 stained 2D gel of total protein from plants at ZT14 (left) and enlarged windows (A-D) of gel (right) shown on left of plants from different time. The numbers assigned to the protein spots correspond to those listed in table 1 and fig. S1.

**Fig. 2**: Functional classification and hierarchical clustering of the identified proteins. **(A)** Functional classification of the identified proteins. **(B)** Hierarchical clustering of the identified protein expression profiles at different times. **(C)** Hierarchical clustering of some important proteins mentioned in fig. 1. Different colors correspond to the proteins’ log-transformed fold-change ratios depicted in the bar at the bottom of the figure.

**Fig. 3**: The number of proteins expressing at peak or trough according to the functional classification in fig. 2 (A) at different times. **(A)** Stress resistance. **(B)** Oxidation reduction processes. **(C)** Energy metabolism. **(D)** Photosynthesis. **(E)** Biosynthesis and metabolism. **(F)** Others.

**Fig. 4**: The change of antioxidant enzyme activities in *Kobresia pygmaea* at different times. Different symbols indicate significant differences between treatments (*P<0.05*) according to Tukey's test.

**Fig. 5**: The effect of environment change on the protein accumulation of abscisic acid synthase and dehydrin for the six samples from different time. The coomassie stained gel is included as a protein loading control.

**Supporting information**

**Table S1**: The dynamic changes of environmental factors at different time when the plant samples were collected.

**Fig. S1**: The first set 2-DE of six samples from different time.

**Fig. S2**: The second set 2-DE of six samples from different time.

**Fig. S3**: The third set 2-DE of six samples from different time.
