# Supplementary material for: Comparative Proteomics Analyses of Kobresia pygmaea Adaptation to Environment along an Elevational Gradient on the Central Tibetan Plateau
Source: PLoS One. 2014 Jun 2;9(6):e98410. doi: 10.1371/journal.pone.0098410 (PMC4041879; doi:10.1371/journal.pone.0098410)
Supplement: File S1 — Supporting Figures. Figure S1, The second set 2-DE of four K. pygmaea samples from different elevations. Figure S2, The third set 2-DE of four K. pygmaea samples from different elevations. (ZIP) [file pone.0098410.s001.zip › Manuscript-1.doc]

**Title:** Comparative proteome analyses reveal that the adaptation strategies of the alpine plant *Potentilla saundersiana* along the altitude gradient in the Northwestern Tibet Plateau

**Author:** Lan Ma^1,2,3^, Xudong Sun^1,2,4^, Yunqiang Yang^1,2,3^, Shihai Yang^3,4^, Xiangxiang Kong^1,2,3^, Xiong Li^1,2,3^, Yongping Yang^1,2,4,*^, Xiangyang Hu^1,2,4,*^

^1^Key laboratory of biodiversity and biogeography, Kunming institute of botany, Chinese Academy of Science, Kunming, Yunnan 650201, China

^2^Plant Germplasm and Genomics Center, the Germplasm Bank of Wild Species, Kunming Institute of Botany, Chinese Academy of Sciences, Kunming, Yunnan 650201, China

^3^University of Chinese Academy of Sciences, Beijing, 100049, China

^4^Institute of Tibetan Plateau Research, Chinese Academy of Sciences, Beijing 100101, China

***Corresponding author**

(X.H.) Email: [huxiangyang@mail.kib.ac.cn](mailto:huxiangyang@mail.kib.ac.cn). Tel: 86-0871-65223069. Fax: 86-0871-835376671. (Y.Y.) Email: [yangyp@mail.kib.ac.cn](mailto:yangyp@mail.kib.ac.cn). Tel: 86-0871-65223234. Fax: 86-0871-83533234.

**Author Email Address:**

Lan Ma: [malan@mail.kib.ac.cn](mailto:malan@mail.kib.ac.cn)

Xudong Sun: [sunxudong@mail.kib.ac.cn](mailto:sunxudong@mail.kib.ac.cn)

Yunqiang Yan: [yangyunqiang@mail.kib.ac.cn](mailto:yangyunqiang@mail.kib.ac.cn)

Shihai Yang: [yangsh@itpcas.ac.cn](mailto:yangsh@itpcas.ac.cn)

Xiangxiang Kong: [kongxiangxiang@mail.kib.ac.cn](mailto:kongxiangxiang@mail.kib.ac.cn)

Xiong Li: [lixiong@mail.kib.ac.cn](mailto:lixiong@mail.kib.ac.cn)

Yongping Yang: [yangyp@mail.kib.ac.cn](mailto:yangyp@mail.kib.ac.cn)

Xiangyang Hu: [huxiangyang@mail.kib.ac.cn](mailto:huxiangyang@mail.kib.ac.cn)

**Running Title:** *P. saundersiana* adaptation to altitude gradient

**Abstract**

The altitude gradient limits the growth and distribution of alpine plants. Multiple environmental stresses along the altitude gradient, including sharp temperature shifts, strong ultraviolet radiation exposure, and low oxygen content, are implicated in this phenomenon. Alpine plants have developed special strategies to survive the chilling environment in high mountains, but the mechanism underlying the evolution remains undefined. The alpine plant *Potentilla saundersiana* is widespread in the Northwestern Tibet Plateau. In this study, we used comparative proteome analyses to investigate the dynamic protein pattern for *P. saundersiana* located at five different altitudes. A total of 118 differentially expressed proteins were successfully detected and functionally characterized. The proteins were members of various functional categories, including material and energy metabolism, antioxidant enzymes, defense response, protein degradation, cell wall synthesis, and hormone signal transduction. Our study confirmed that increasing levels of antioxidant proteins, and their respective activities, and accumulation of primary metabolites, such as proline and sugar, confer *P. saundersiana* tolerance to the alpine environment. The epigenetic regulation of DNA stability and post-translation regulation of protein degradation also play important roles in the development of tolerance to environmental stress. Our results also showed that *P. saundersiana* modulated root architecture and leaf phenotype to enhance adaptation to alpine environmental stress through hormone synthesis and signal transduction, particularly the cross-talk of auxin and strictosidine. Based on these findings, we conclude that *P. saundersiana* uses multiple strategies to adapt to the high-altitude environment in the Tibet plateau.

**Introduction**

Most alpine plants confront harsh climates in the high mountain altitudes, including extreme low temperatures, dryness, low oxygen concentration, and exposure to strong ultraviolet (UV) radiation and violent wind. Therefore, alpine plants have evolved to include several defense mechanisms to survive under these conditions. For example, most alpine leaves and stems are covered with floss to protect them from stress caused by low environmental temperatures. Plants can also avoid damage caused by freezing or ice crystallization within plant tissues by increasing the amount of solutes in their tissues, such as anti-freezing protein and proline.^1-3^ Plants can avoid water loss by deepening root structures and increasing stomatal control. Some alpine plants, such as succulent plants, often use crassulacean acid metabolism (CAM) photosynthesis in [arid](http://en.wikipedia.org/wiki/Arid) conditions.^4^ Morphologically, alpine plants exhibit dwarfism with a small leaf index, which avoids stress caused by strong wind.^1,2^ Most flowers of alpine plants are blue, which avoids exposure to UV radiation. Environmental stress normally causes the quick accumulation of reactive oxygen species (ROS), which trigger plant defense responses to environmental stress.^4,5^ However, the over-accumulation of ROS damages the plant tissue; most alpine plants are able to activate both enzymatic and non-enzymatic antioxidants, such as superoxide dismutase (SOD), catalase (CAT), ascorbate peroxidase (APX) and glutathione reductase (GR), to efficiently scavenge ROS and avoid over-accumulation.^6^ Early signals, such as the calcium burst and the downstream kinase cascade pathway, are also important for plant response to environmental stress.^7^ Increasing evidence supports the hypothesis that post-transcriptional modification and epigenetic modulation are also involved in plant stress response.^8-10^ At the morphological level, alpine plants use several strategies to adapt to the high-altitude environment, but the underlying molecular and physiological mechanisms of the adaptation remains undefined.^5,11^

The alpine plant *Potentilla saundersiana* is a common species distributed throughout alpine meadows in Northern China that demonstrates tolerance to UV exposure and low temperature stresses.^12^ It is widespread along the alpine altitude gradient, which ranges from 3500 to 5500 m, indicating its successful acclimation to the alpine environment. To understand its integral adaptation mechanisms, we collected five sets of *P. saundersiana* samples along the altitude gradient in the high mountains of the Northwestern Tibet Plateau. We combined physiological and proteome analyses to evaluate the plant’s responses to altitude gradient differences. We identified several differentially expressed proteins, which were isolated by comparative proteome analyes, that are involved in the antioxidant system, early signaling events, the protein kinase pathway, and protein phosphorylation. Further analysis demonstrated that these proteins provide the basis for the physiological adaptation of *P. saundersiana* located at different altitudes. Based on these results, we hypothesized that the alpine plant *P. saundersiana* uses multiple strategies to acclimate to alpine environmental stress.

**Materials and Methods**

*Experimental sample collection*

This study was conducted in mid-July, 2012, on the south-facing slope of the Nyainqentanglha Mountains from 4350 to 5250 m in altitude (30°30′–30°32′N, 91°03′E) near Damxung county in central Tibet. The summit of the slope is at an elevation of approximately 5600 m. We selected one site approximately every 100 to 200 m in the altitude along the transect from 4350 to 5250 m for a total of 5 sample sites: 4350 m, 4600 m, 4900 m, 5100 m, and 5200m. We collected the samples at each location at 10am on sunny days to avoid weather inference. At each site, a total of 100 to 200 fully expanded and intact leaves from a 10-m^2^ region were randomly selected and immediately frozen by liquid nitrogen for later protein extraction and enzyme analysis. The samples were collected and the experiments were conducted in triplicate.

*Specific leaf index and leaf nitrogen concentration (Nmass) measurements*

At each site, 200 to 400 fully expanded and intact leaves were randomly selected. The specific leaf index (SLA) and the rate of photosynthesis were measured as previously described.^13,14^

*Histology and microscopy*

In order to visualize lignified cell walls in the leaves, methanol-cleared leaves were examined for lignin autofluorescence and collection of differential interference contrast (DIC) images under a microscope. Briefly, the plant leaves from different altitudes were washed with methanol and quickly fixed in 2% formaldehyde and embedded in low-viscosity (Spurr’s) resin (Electron Microscopy Sciences, Pennsylvania, USA). One micrometer-thick sections from embedded tissues were cut with a microtome, stained with toluidine blue or 0.01% calcofluor white, and observed with a UV fluorescent microscope as previously described.^15^

In order to evaluate the stomatal shape and size, environmental scanning electron microscopy was conducted using an FEI QUANTA 200 3D equipped with a large-field secondary electron detector (SED) and operated at 20 kV. Fresh specimens were fixed in formaldehyde:acetic acid:ethanol (FAA) at 4°C for 2 h. The samples were then dehydrated through a graded ethanol series of 70, 85, 95, and 100% ethanol, each for 45 min; the 100% ethanol was changed once. Specimens were critical-point dried using liquid carbon dioxide and then coated with gold and palladium.^16^

*Analysis of anthocyanin and proline contents*

Total fresh leaves (1 g) were ground using a mortar and pestle with a small amount of quartz sand. Next, 1% HCl/menthone (for anthocyanin) was added and the samples were ground until the sample turned white. The samples were transferred to a 25-mL container, and the mortar and pestle were repeatedly washed. The final volume of each sample was adjusted to 25 mL using 80% acetone and then it was filtered. Anthocyanin contents were measured at absorbances of 470, 548.6 and 664.2 nm (for chlorophyll) using a spectrophotometer (Bio-Rad, California, USA).^17^ The proline content was measured as previously described.^18^

*Antioxidant enzyme activities assay*

To measure the antioxidant enzyme activities, 5 g of leaf sample was homogenized to a fine powder with a mortar and pestle under liquid nitrogen. Then, the powder and pestle were ground with 30 mL of extraction buffer containing 50 mM sodium phosphate buffer (pH 7.0), 0.2 mM EDTA and 2% polyvinylpolypyrrolidone (PVPP) for 10 min. The homogenates were ﬁltered through two layers of cheesecloth and centrifuged at 4°C at 15000×g for 15 min. Supernatants were desalted on a Sephadex G-50 column and this final sample was used to measure the enzymatic antioxidant activities. The activities of APX and SOD were measured spectrophotometrically by monitoring the change in A290 and A560, respectively.^19,20^

*In-gel enzyme activity staining*

Equal amounts of protein from each leaf sample were loaded on to a discontinuous polyacrylamide gel electrophoresis (PAGE) gel under nondenaturing conditions (4°C, 4 h, 35 mA). Native-PAGE activity staining was performed on 10% (w/v) acrylamide slab gels and the in-gel activity staining of SOD and APX were detected as previously described.^19,20^

*H_2_O_2_ and O_2_^-^ content measurements*

To measure hydrogen peroxide (H_2_O_2_) content, approximately 10 g of leaf samples from each of the different altitudes were collected and quickly frozen by liquid nitrogen. The samples were then homogenized with extraction buffer (Tris-HCl buffer, 10 mM, pH 6.0) and the supernatants were obtained by centrifugation at 4°C at 15000×g for 15 min. An equal volume of reaction buffer (0.25 mM FeSO_4_, 0.25 mM (NH_4_)_2_SO_4_, 25 mM H_2_SO_4_, 1.25 μM xylenol orange, and 1 mM sorbitol) was added to each sample at room temperature for 1 h. Absorbance was measured at 560 nm, and the H_2_O_2_ level was calculated as previously described.^21^

The content of superoxide anion (O_2_^-^) was measured by chemiluminescence in the presence of the chemiluminogenic probe lucigenin. Briefly, a sample of approximately 1 g was extracted with 5 mL of extraction buffer (10 mM Tris-HCl, 10mM CaCl2, 20 mM NaCl, 10 mM KCl, pH 8.0), and the supernatant was obtained by centrifugation at 4°C at 15000×g for 15 min. The O_2_^-^ content was immediately measured using the superoxide anion detection kit (Cat. 574590, Merck, Germany) according to the manufacturer’s recommended protocol.

*Auxin and strigolactone content measurements*

To measure the auxin content, 500 mg of plant leaf sample was homogenized in liquid nitrogen with 1 ml of extraction buffer (10 mM Na-phosphate buffer, pH 7.0, 0.02% w/v of sodium diethyldithiocarbamate, 50 pg [^13^C_6_]IAA (>99%) as an interna1 standard), and the sample was extracted under continuous shaking for 1 h at 4°C. After extraction, the pH was adjusted to approximately 2.6 with 1 M HC1, and the sample was slurried with 35 mg of Amberlite XAD-7 (Sigma-Aldrich, Missouri, USA) for 30 min. After removal of the buffer, the XAD-7 was washed with 1 mL of 1% acetic acid before being slurried with 1 mL of dichloromethane for 30 min. The combined dichloromethane fractions were reduced to dryness in a Speed Vac concentrator (Savant Instruments, Inc., New York, USA). The content analysis of auxin was manipulated as previously described.^22^

To measure the strigolactone content, 5 g of plant leaf sample was extracted twice with two volumes of ethyl acetate. The ethyl acetate phase was washed with 0.2 M K_2_HPO_4_, dried over anhydrous Na_2_SO_4_, and concentrated in a Speed Vac concentrator. The extracts were dissolved in 50% (v/v) acetonitrile and were subjected to LC/MS-MS analysis using a triple quadruple tandem mass spectrometer (Xevo TQ-S; Waters MS Technologies, Massachusetts, USA) and Ultra Performance Liquid Chromatograph (Acquity UPLC; Waters) equipped with a reverse phase column (BEH-C18; Waters). The mobile phase was changed from 30% (v/v) acetonitrile to 40% and 70% (v/v) linearly at 6 and 15 min after the injection, respectively, at a flow rate of 0.4 mL min^-1^. The column temperature was set to 25°C. MS parameters were set to the following values: desolvation gas flow, 800 L·h^-1^; capillary voltage, 3800 V; cone voltage, 30 V; desolvation temperature, 350°C; source temperature, 120°C; and collision energy, 15 V; and using MRM 331.16 > 216.10 transition for the epi-5DS detection. Five pg/mL epi-5DS in 50% (v/v) acetonitrile was used as a reference.^23^

*Protein extraction and 2D electrophoresis*

Protein extraction and 2D separation were performed according to a previously reported method, with minor medications.^19^ Approximately 10 to 20 g of the treated leaves was ground in liquid nitrogen and the total soluble protein was extracted at 4°C in 5 mL of 50 mM Tris-HCl buﬀer (pH 7.5) containing 20 mM KCl, 13 mM DTT, 2% (v/v) NP-40, 150 mM PMSF and 1% (w/v) PVPP. The homogenates were centrifuged (12000×g, 15 min, 4°C) and the supernatants were added to ﬁve volumes of acetone containing 10% (w/v) TCA and 1% (w/v) DTT. The samples were maintained at -20°C for 4 h and then centrifuged (25000×g, 30 min, 4°C). The resulting pellets were washed with acetone containing 1% (w/v) DTT at -20°C for 1 h and then centrifuged; the wash step was repeated. The ﬁnal pellets were vacuum-dried and dissolved in 8 M urea, 20 mM DTT, 4% (w/v) CHAPS and 2% (w/v) ampholyte (pH 3−10). The samples in ampholyte were vortexed thoroughly for 1 h at room temperature and then centrifuged (25000×g, 20 min, 20°C) and the supernatants were collected for 2D electrophoresis (2DE). Each experiment was repeated three times. Extracted proteins were ﬁrst separated by isoelectric focusing (IEF) using gel strips to form an immobilized nonlinear pH gradient from 4 to 7 (Immobiline DryStrip, pH 4−7 NL, 17 cm; Bio-Rad) and then by SDS−PAGE using 12.5% polyacrylamide gels. The strips were rehydrated for 16 h in 450 μL of dehydration buﬀer containing 800 μg of total proteins and a trace of bromophenol blue. The strips were focused at 20°C for a total of 64 kV/h using the PROTEAN IEF system (Bio-Rad). After IEF, the strips were equilibrated for 15 min in equilibration buﬀer (6 M urea, 0.375 M Tris, pH 8.8, 2% (w/v) SDS, 20% (v/v) glycerol and 2% (w/v) DTT). For 2D SDS-PAGE, the strips were placed on top of 12.5% (w/v) SDS-PAGE gels. Gel electrophoresis was performed at 25 mA for 5 h. The gels were stained using the colloidal CBB staining method. After staining, the gels were scanned using the GS-800 Calibrated Densitometer (Bio-Rad) and PDQUEST software (Bio-Rad), based on their relative volumes. Parameters were optimized as follows: saliency, 2.0; partial threshold, 4; and minimum area, 50. Spots were quantiﬁed by determining the ratio of the volume of a single spot to the all the spots on the gels. The relative volume of each spot was assumed to represent its expression level. A criterion of p<0.05 was used to deﬁne a signiﬁcant diﬀerence when analyzing the parallel spots between different treatments with one-way ANOVA using SPSS software (<http://spss.en.softonic.com/>). To compensate for subtle differences in sample loading or gel staining/destaining during individual repeat experiments, the volume of each spot was normalized. Each experiment was repeated three times.

*In-gel digestion*

Protein spots that showed signiﬁcant changes in expression during the treatments were excised manually from colloidal CBB-stained 2DE gels. Protein digestion with trypsin was performed as follows. Individual spots of interest were excised from the 2DE gels using sterile tips and placed in 1.5 mL sterile tubes. Each polyacrylamide spot was destained with 50 mM NH4HCO3 for 1 h at 40°C, then reduced with 10 mM DTT in 100 Mm NH_4_HCO_3_ for 1 h at 60°C and incubated with 40 mM iodoacetamide in 100 mM NH_4_HCO_3_ for 30 min. The gel pieces were minced and allowed to dry, and then rehydrated in 12.5 ng/μL trypsin (sequencing grade; Roche Diagnostics, Indiana, USA) in 25 mM NH_4_HCO_3_ at 37°C overnight. The trypsin peptides were extracted from the gel grains with 0.1% triﬂuoroacetic acid in 50% acetonitrile three times. Supernatants were concentrated in a Speed Vac concentrator to approximately 10 μL and desalted using Zip-Tips (C18resin, P10; Millipore Corporation, Massachusetts, USA). Peptides were eluted from the column with 50% acetonitrile/0.1% triﬂuoroacetic acid. The protein spots that changed more than 1.5-fold and passed the Student’s t-test (p<0.05) were selected and identified by mass spectrometry (MS) analysis.

*MALDI-TOF/TOF analysis and database search*

The lyophilized peptide samples were dissolved in 0.1% trifluoroacetic acid (TFA). MS analyses were conducted using a MALDI-TOF/TOF mass spectrometer 4800-plus Proteomics Analyzer (Applied Biosystems; Massachusetts, USA). MS acquisition and processing parameters were reﬂector positive mode and 800−3500 Da acquisition mass range. The laser frequency was 50 Hz, and 700 laser points were collected for each sample signal. For each sample, 4 to 6 ion peaks with signal-to-noise ratios greater than 100 were selected as precursors for secondary MS analysis; the TOF/TOF signal for each precursor was assessed with 2000 laser points. The primary and secondary MS data were transferred into Excel ﬁles as inputs to search against an NCBI nonredundant database; the search was restricted to viridiplantae (green plants) using the MASCOT search engine (www.matrixscience.com). The search parameters were established as follows: no restriction of protein molecular weight; one missed trypsin cleavage allowed; cysteine treated by iodoacetamide; and oxidation of methionine. The peptide tolerance was 100 ppm and the MS/MS tolerance was 0.25 kD. Protein identiﬁcations were validated manually, with at least 4 peptides matching. The keratin contamination was removed and the MOWSE score threshold was greater than 60 (p<0.05). According to the MASCOT probability analysis, only signiﬁcant hits were accepted for the identiﬁcation of the protein sample.

*Western blotting analysis*

SDS-PAGE was performed as described by Laemmli using 12% (w/v) polyacrylamide slab gels. For Western blot analysis, the protein samples were electroblotted onto polyvinylidene diﬂuoride (PVDF) membranes using a Trans-Blot cell (Bio-Rad). After transfer, the membranes were probed with the appropriate primary antibodies and HRP-conjugated goat antirabbit secondary antibody (Promega, Wisconsin, USA) and the signals were detected using an ECL kit (GE Company, Indiana, USA). The primary antibodies (all obtained from Agrisera, Inc., Vanna, Sweden) were diluted as follows: polyclonal antibody against plant HSP (1:3000), dehydrin (1:2000), Atg8a (1:1000), SOD (1:2000), APX (1:1000) and actin (1:3000).

**Results and Discussion**

*Physiological and morphological differences for* P. saundersiana *distributed along the altitude gradient*

The alpine plant *P. saundersiana* is widely distributed in the Northwestern Tibet Plateau. To investigate the effects of the altitude gradient on the growth and development of *P. saundersiana*, we first measured the soil temperature and rainfall at different altitudes during the annual growing season for *P. saundersiana*, which normally takes place during July. The soil temperature significantly decreased (p<0.05) and rainfall increased with increasing altitude (Supplemental Figure 1). The PCA analysis of the effects of different factors, including soil temperature, humidity, average sunlight time, average rainfall, and average UV radiation exposure, indicated that the main component contributed 67.5% towards adaptation. Soil temperature and average rainfall were the primary factors in the main component, suggesting that soil temperature and rainfall (or soil water content) are two factors that limit alpine *P. saundersiana* growth and distribution (Supplemental Figure 1). UV radiation also contributed (0.774) to the main component, but not as much as soil temperature (-0.938) and rainfall (0.906). Therefore, we mainly considered the effects of temperature and waterfall on *P. saundersiana* adaptation along the altitude gradient in our experiments.

We compared the phenotypic differences of *P. saundersiana* at different altitudes. As shown in Figure 1A&B, we observed distinct differences in the root system architecture. Plants located below an altitude of 4900 m have an independent main root system for each plant, but, at higher altitudes, the underground main root of two or more plants joined together, which might assist the plant’s adaptation to harsh environments at higher altitudes. Dwarfism of the plants gradually appeared with increasing altitude. We could not identify a difference among the three leaf samples from altitudes of 4350 m, 4600 m and 4800 m, but the leaves were smaller at altitudes of 5100 m and 5200 m (Figure 1C). The stomatal aperture increased at altitudes from 4350 m to 4800 m; it reached its maximum level at an altitude of 4800 m and then decreased with further increases in altitude (Figure 1D, Figure 2A). The SLA and Nmass are two important criteria to evaluate alpine plant adaptation to environmental stress, and we found that SLA and Nmass gradually increased as the altitude increased. They reached their peak levels at 5100 m and sustained the increase at 5200 m. A similar pattern was observed for the Fv/Fm ratio, which reflected the efficient photosynthesis capability of the plant (Figure 2).

*Dynamic protein profiling in the leaves of* P. saundersiana *distributed along the altitude gradient*

To define the underlying adaptation mechanism of *P. saundersiana* to the altitude gradient, we applied a comparative proteomic approach for protein identification and quantification. A total of 118 protein spots were successfully quantified with significant differential expression changes among the leaf samples from five different altitude locations (4350 m, 4600 m, 4800 m, 5100 m, and 5200 m). All of the proteins demonstrated increased expression (>2) or decreased expression (<0.5) at high altitudes (4600 m, 4800 m, 5100 m, and 5200 m) compared to samples from the lowest altitude (4350 m), which was used as the control (Supplemental Table 1, Supplemental Figure 3, Table 1). The identified proteins were divided into nine groups based on their biological functions (Figure 3, Figure 4A). The majority of these proteins were sorted one of three categories: general energy and metabolism, protein kinase and phophosatase activity, and cell structure and division proteins. A hierarchical cluster analysis was conducted to categorize the proteins that showed differential expression profiles in response to the altitude gradient (Figure 4B&C). Most proteins were antioxidant enzymes, defense response proteins, and cell wall-related proteins; cell structure and division proteins were clustered together (Figure 4C). In addition, the proteins involved in hormone synthesis and response were up-regulated with increasing altitude, suggesting the important roles of hormone synthesis and metabolism in the acclimation of the alpine plant *P. saundersiana* to environmental altitude gradients.

*Material and energy-associated proteins confer plant acclimation to higher-altitude environments*

Plants located in higher altitudes require more energy to respond to serious environmental stress.^3^ We observed that the activities of most proteins related to material and energy metabolism changed dramatically at high altitudes. The carbohydrate is the main nutrient for supplying organs with energy, and, in our experiments, the proteins involved in reducing or non-reducing sugar synthesis, including soluble starch synthase (spot 7), fructan 1-exohydrolase (spot 11), fructose-1, 6-bisphosphatase (spot 41), and glyceraldehyde-3-phosphate dehydrogenase (spot 105), were differentially up-regulated with increasing altitude, suggesting the important roles of reducing and non-reducing sugars in modulating plant acclimation to alpine environments. Meanwhile, we analyzed different reducing sugar and total sugar contents in the plant samples distributed along the altitude gradient. The level of reducing sugars, such as glucose and fructose, and non-reducing sugars, such as disaccharose, were higher in the plants located at high altitudes; these levels reached their maximums at altitudes of 5100 m and 5200 m. We also measured the soluble sugar content in the samples from different altitudes, and we observed that increasing altitude caused an increase in soluble sugar content of the plant, suggesting the important role of sugar accumulation in alpine plant tolerance to environmental stress (Figure 5A). Recently, Wang et al reported that starch and sucrose metabolism is pivotal for salt tolerance in the halophyte plant *Thellungiella halophile*,^24,25^ which is in agreement with our results. Meanwhile, beta-glucosidase 22 (spot 14), alpha-amylase isozyme 3E (spot 20), glucan endo-1,3-beta-glucosidase (spot 24), and fructose-1,6-bisphosphatase (spot 40) show differential down-regulation with increasing altitude, indicating the complicated regulation mechanisms of carbon metabolism in plant response to alpine environmental stress.

Trehalose is a non-reducing disaccharide present in diverse organisms, and it is quickly accumulated in response to environmental stresses, including drought and exposure to salt and cold. The transgenic *Arabidopsis* that overexpresses microbial trehalose biosynthesis genes displays tolerance to multiple abiotic stressors.^26^ Trehalose-6-phosphate, via trehalose-phosphate synthase, is the precursor of trehalose. Trehalose-phosphate synthase (spot 5) increased in plants in higher altitudes, which supports the association of plant tolerance to environmental stress at high altitude.

Proline is an important osmolyte in plants that functions in stress adaptation.^27,28^ ∆^1^pyrolline-5-carboxylate-synthetase (P5CS), which catalyzes the rate-limiting step of proline biosynthesis from glutamate, is up-regulated in response to environmental stress in plants. Pyrroline-5-carboxylate synthase (spot 2) was up-regulated with increasing altitude. Further, the proline content also gradually increased in *P. saundersiana* along the altitude gradient (Figure 5B), suggesting the role of proline metabolism in alpine plant adaptation to environmental stress.

Spermidine synthase, arginase and ornithine decarboxylase mediate the metabolism of spermine and nitric oxide (NO), both of which are essential for modulating plant tolerance to abiotic stresses, including cold, drought and exposure to UV radiation.^27,29^ Spermidine synthase 2 (spot 40), arginase (spot 103) and ornithine decarboxylase (spot 111) were up-regulated with increasing altitude, suggesting a role of spermine and NO in alpine plant adaptation to high altitude environments. Allene oxide cyclase is believed to be one source of jasmonic acid (JA) synthesis in plants. The novel JA is an important hormone for plant defense response.^30^ Heme oxygenase mediates carbon monoxide synthesis, and carbon monoxide can affect NO function during plant response to salt and drought stress.^31^ Heme oxygenase 1 (spot 79) and allene oxide cyclase (spot 84) were up-regulated with increasing altitude. Possibly, both enzyme activities increase to promote plant adaptation to alpine environments through modulating NO synthesis.

Glutamine synthetase (GS) is not only the key enzyme for plant ammonia assimilation, but also plays a significant role in plant stress resistance. Cysteine synthase, as part of cysteine synthesis, regulates plant tolerance to abiotic stress by modulating glutathione (GSH) content and cytosolic redox status.^32^ GS cytosolic isozyme 1-2 (spot 57) and cysteine synthase (spot 58) were up-regulated with increasing altitude. However, a different isozyme of GS, glutamine synthetase cytosolic isozyme 1-5 (spot 105), and glutamate dehydrogenase 1 (spot 115) were down-regulated with increasing altitude, suggesting the complicated regulation of glutamine for alpine plant response to environmental stress.

The first committed step of anthocyanin biosynthesis is the formation of chalcone, catalyzed by chalcone synthase (CHS).^33,34^ We observed a gradual accumulation of anthocyanin content in plants with increasing altitude. Anthocyanin content reached its maximum level at 5100 m and 5200 m (Figure 5B), suggesting a role of anthocyanin for plant tolerance to alpine environmental stress. Accordingly, chalcone synthase D (spot 112) was up-regulated in plants with increasing altitude. Flavonoid accumulation in plants can be induced by biotic and abiotic stresses, such as UV-B radiation exposure, drought or low temperatures.^35^ Flavonoid 3'-O-methyltransferase 1 (spot 51) and isoflavone reductase (spot 65) were differentially up-regulated with increasing altitudes. Anthocyanin is the main component of plant flavones, and increasing altitude also caused an increase in anthocyanin content (Figure 5B), suggesting an important role of flavone metabolism in plant tolerance to environmental stress.

*Antioxidant enzyme systems prevent ROS over-accumulation in the alpine plant*

The GSH-ascorbate cycle primarily detoxifies ROS, since over-accumulation of ROS causes irreversible damage to the alpine plant.^6,32^ A consistent increase of GSH-S-transferase (spot 76), specifically L-ascorbate peroxidase 1 (spot 88), indicates that the GSH-ascorbate cycle actively participates in maintaining the redox status to an appropriate level in plants at high altitudes. Cystathionine gamma-synthase (spot 107) and monodehydroascorbate reductase (spot 109) are the primary enzymes for the GSH-ascorbate cycle and were significantly up-regulated in the plants located at 5100 m and 5200 m, compared to samples at lower altitudes. Thioredoxin is a protein that acts as an antioxidant by facilitating the reduction of other proteins by cysteine thiol-disulfide exchanges; it plays a key role in the maintenance of cellular redox homeostasis through the sensing and transferring of reducing equivalents. Thioredoxin also plays an important role as a signaling intermediate that senses the redox state and transmits the redox signal to other components in chloroplasts, mitochondria and the cytosol. Thioredoxin domain-containing protein 9 (spot 96) was up-regulated with increasing altitude, suggesting a possible role for thioredoxin in plant adaptation to high-altitude environmental stress.

Both plant peroxidases and superoxide dismutase are important in diverse cellular functions, such as lignin biosynthesis, hormone generation and detoxification of hydrogen peroxide. In our experiments, peroxidase 36 (spot 42) and superoxide dismutase (spot 75) were up-regulated with increasing altitude, suggesting their roles in scavenging ROS in plants at high elevations. Further, we observed an increase of ROS, including H_2_O_2_ and O_2_^-^, with increasing altitude, and ROS levels reached their maximums at 5100 m and 5200 m (Figure 5C). We also observed increased activities of antioxidant APX and SOD enzymes in samples from higher altitudes (Figure 5D). The higher antioxidant enzyme activities in the samples from high altitudes can efficiently scavenge the over-accumulation of H_2_O_2_ and O_2_^-^ to protect the plant from ROS damage. These results agree with a previous report that the plant redox system plays an important role in plant adaptation to environmental stress.

*Thick cell wall structure increases alpine plant adaptation to environmental stress*

The plant cell wall is the first mechanical barrier preventing the cell from pathogen attack or environmental stress,^36^ but the expression of cell wall biosynthesis proteins is affected by environmental conditions or pathogen-related stresses.^37^ Plant cell walls can be classified into three basic types: parenchyma, collenchyma, and sclerenchyma. Parenchyma and collenchyma cells have only primary walls, and sclerenchyma cells have both primary and secondary walls. The synthesis of primary and secondary cell walls could be modulated by genetic and environmental factors. We observed different cell wall structures in leaf samples from high altitudes. As shown in Figure 6, the cell wall thickness gradually increased with increasing altitude. The cell walls were thickest at altitudes of 5100 m and 5200 m, and the cell walls were thinner in the leaves from 3600 m and 3800 m altitudes. We also observed bigger vessels and phloem in the leaves from the higher altitudes (5100 m and 5200 m) compared to samples from lower altitudes (3600 m and 3800 m). The cortex cell was also bigger in the leaves from the higher altitudes than those from the lower altitudes. Consistent with the cell wall structure, the proteins involved in cellulose and lignin synthesis, including xylan-1,4-beta-xylosidase (spot 4), cellulose synthase (spot 22) and caffeoyl-CoA 3-O-methyltransferase (spot 90), were up-regulated with increasing altitude, which might affect cell wall synthesis and, ultimately, the thickness of the cell wall. Expansin protein is a nonezymatic protein found in the plant cell wall, and it has important roles in plant cell growth and development.^38^ Expansin-like A4 (spot 98) was significantly up-regulated with increasing altitude, which might contribute to cell wall synthesis in alpine environments. Actin is a globular multi-function protein that forms microfilaments. Actin (spot 19) and actin-related protein 7 (spot 29) were down-regulated with increasing altitude, suggesting that alpine plants organize microfilament synthesis to adapt to environmental stress.

*The role of protein kinase and phosphatase in alpine plant adaptation to environmental stress*

Protein phosphorylation and dephosphorylation are basic post-translation modifications that modulate plant response to environmental stress.^39^ Serine/threonine-protein kinase SAPK10 (spot 44), mitogen-activated protein kinase kinase 1 (spot 53), mitogen-activated protein kinase kinase 6 (spot 106), mitogen-activated protein kinase 11 (spot 114), mitogen-activated protein kinase homolog NTF3 (spot 116), and protein phosphatase 2C (spot 110) were differentially regulated with increasing altitudes, which demonstrates the complex regulation mechanisms for alpine plant response to environmental stress. Hexokinase is an important kinase for glucose signal transduction,^40^ and hexokinase-2 (spot 5) was up-regulated with increasing altitude, suggesting an important role for glucose signaling, or glucose as the reducing sugar, in alpine plant adaptation to environmental stress. This finding further supports previous reports that reducing sugar improves plant response to environmental stress.

Calcium signaling initiates plant response to multiple environmental stressors by activating the downstream calcium-dependent kinase cascade.^28^ Calcium-dependent protein kinase SK5 (spot 13) was differentially regulated, suggesting different roles for calcium signaling and its downstream kinase cascade in plant response to environmental stress.

*Differential expression pattern of transcriptional factors and defense response-related proteins in the alpine plant along the altitude gradient*

A series of defense-related transcriptional factors were differentially expressed in the plant samples from high altitudes.^1,3,28^ The heat stress transcription factor A-1 (spot 16) was up-regulated, while the heat stress transcription factor A-9 (spot 520) was down-regulated, with increasing altitude. Both of these transcriptional factors were affected by high temperature, and it is possible that they were also affected by low temperature stress in a high-altitude environment. Other transcriptional factors, including WRKY transcriptional factor (probable WRKY transcription factor 10, spot 31) and MYB transcriptional factors (transcription factor MYB12, spot 48; transcription factor MYB28, spot 55; myb-related protein Hv33, spot 70), were differentially regulated with increasing altitude, suggesting their different roles in modulating alpine plant response to environmental stress. This finding agrees with previous reports that WRKY and MYB transcriptional factors play multiple roles in tolerance to environmental stress by activating or suppressing corresponding target protein transcription.

Several defense-related proteins were modulated to enhance plant tolerance to alpine environmental stress.^3^ Cold- and drought- related proteins, including low-temperature-induced protein (spot 8), heat shock 70 kDa protein 18 (spot 12), dehydrin COR410 (spot 72) and 17.7 kDa class I heat shock protein (spot 86), were up-regulated with increasing altitude, suggesting their important roles in alpine plant acclimation to environmental stress. Desiccation-related protein PCC13-6 (spot 61) was down-regulated with increasing altitude, suggesting that drought stress is not a major environmental factor that limits growth for the alpine plants at high altitudes.

Consistent with the above data, our western blotting results confirmed that the proteins corresponding to HSP70, dehydrin, APX and SOD were up-regulated with increasing altitude (Figure 7A).

*The novel role of protein degradation or autophagy in plant tolerance to alpine environmental stress*

In order to maintain cellular homeostasis, the amount and quality of proteins in cells is strictly controlled, not only by protein synthesis, but also by protein degradation. The ubiquitin-proteasome pathway is essential for multiple physiological systems through selective degradation of target proteins.^41,42^ The proteins designated for proteasome-mediated degradation are conjugated with the polypeptide ubiquitin, which is then targeted to the 26S proteasome complex. In addition to ubiquitin, there are several ubiquitin-like proteins (UBLs) that can also be conjugated to substrate proteins and alter their respective functions. Specifically, the small ubiquitin-related modifier (SUMO) covalently modifies a large number of proteins with important roles in many cellular processes, including gene expression, chromatin structure, signal transduction, and genome maintenance.^43^ Environmental stress also affects dynamic changes in proteins by modulating ubiquitin-proteasome or SUMO-proteasome pathways. Interestingly, we found that E3 ubiquitin-protein ligase RING1-like protein (spot 35), E3 ubiquitin-protein ligase SINA-like 5 (spot 43), ubiquitin-conjugating enzyme E2-23 kDa (spot 81), SUMO-activating enzyme subunit 1B-1(spot 68), and 26S protease regulatory subunit 7(spot 113) were differentially up-regulated in plants from higher altitudes.

Autophagy, a process in which cytoplasmic components are taken up into a vacuole or lysosome for protein degradation, is another major proteolytic system that degrades proteins.^44^ Degradation of oxidized proteins by the autophagy process have been reported in *Arabidopsis*.^45^ Autophagy protein 5a (spot 27) was significantly up-regulated in plants from higher altitudes, which was confirmed by western blotting results (Figure 7A). These findings support the role of autophagy in the degradation of oxidized proteins in plants at high altitudes.

*Modulating DNA stability to enhance alpine plant tolerance to environmental stress*

Environmental stress causes DNA damage, but there are many mechanisms to avoid or repair the damage so organisms are able to survive the stress.^46,47^ In our plant samples, DNA repair protein RAD23 (spot 32) was up-regulated with increasing altitude, suggesting that stabilizing the DNA structure by DNA repair proteins enhances plant tolerance to environmental stress. SWI/SNF is a nucleosome remodeling complex found in both eukaryotes and prokaryotes. It expresses DNA-stimulated ATPase activity and destabilizes the histone-DNA interaction in reconstituted nucleosomes in an ATP-dependent manner.^48^ The protein-encoding SWI/SNF complex subunit SWI3A (spot 21) was up-regulated, suggesting an important role for epigenetic regulation in alpine plant response to environmental stress.

*Differential hormone synthesis and response modulate alpine plant adaptation to environmental stress*

Plant hormones, including ethylene, gibberellin acid (GA) and abscisic acid (ABA), and their respective signal transduction and response pathways, participate in plant response to multiple environment stresses. The ABA receptor PYL8 (spot 92) and the GA receptor GID1A (spot 142) were up-regulated with increasing altitude. At the same time, downstream hormone response proteins, including ethylene insensitive 3-like 2 protein (spot 6), DELLA protein RGA2 (spot 10), ethylene-responsive transcription factor ERF060 (spot 37), and ABA-responsive protein ABR17 (spot 82), showed different degrees of up-regulation, suggesting an important role for these hormones in alpine plant adaptation to environmental stress.

Plant root architecture is adjusted in response to environment stress.^1,3^ The above-ground stems showed more branches for the plants at an altitude of 5200 m, and the branch number decreased in the plant stems at the lower altitude of 3600 m. The stem diameter was wider for the plants at the low altitude. At the higher altitude, two or more above-ground plants shared the same underground main root. This phenotype was infrequent for plants below 5200 m, suggesting that such a special phenotype is more advantageous for environmental stress at high altitudes. Proteomics results showed that auxin synthesis and signal transduction related proteins, including auxin response factor 8 (spot 1), indole-3-acetic acid-amido synthetase GH3.5 (spot 3), IAA-amino acid hydrolase ILR1-like 4 (spot 56), and auxin-responsive protein IAA19 (spot 77), showed a higher level of accumulation for the samples at high altitudes of 5100 m and 5200 m. The same compounds showed low levels of accumulation for the samples at lower altitudes, suggesting a role of auxin synthesis or signal transduction for plant adaptation to high altitude environmental stress. We also measured the auxin content in plants at different altitudes (Figure 7B), and we found that auxin content gradually increased with increasing altitude, which is consistent with the proteomics analysis. As previously reported, the high level of auxin in the high-altitude samples could enhance plant tolerance to abiotic stress, such as cold tolerance.^49^ Interestingly, strictosidine synthase (spot 69) was down-regulated in the plant samples at the high altitudes of 5100 m and 5200 m. Strictosidine is a novel plant hormone that modulates plant branching by interacting with auxin signals; we measured the content of strictosidine and found that increasing altitude resulted in decreased strictosidine content (Figure 7C). Previous reports showed that strictosidine suppresses the effect of auxin signals to prevent adventitious root formation.^50^ Our results also showed that increasing the altitude increased auxin content. We also observed fewer lateral roots and smaller root architecture with increasing altitude, which coincides with the previous study that reported that strictosidine and auxin show different roles in regulating root and shoot development.

Similar to the auxin-related protein pattern, proteins related to ethylene and gibberellins synthesis and signal transduction, including ethylene insensitive 3-like protein (spot 6), DELLA protein RGA2 (spot 10), ethylene-responsive transcription factor ERF060 (spot 37), and ABA-responsive protein ABR17 (spot 82), were up-regulated with increasing altitude, suggesting the roles of these hormone response and signal transduction proteins in alpine plant adaptation to environmental stress. Interestingly, the ABA receptor protein (abscisic acid receptor PYL8, spot 92), and gibberellin receptor protein (gibberellin receptor protein GID1A, spot 104), were also up-regulated at high altitudes, indicating alpine plant adaptation to environmental stress through modulating upstream hormone receptor content and perceiving, not simply controlling, downstream hormone signal transduction.

**Conclusion**

In this study, we adopted a comparative proteomics approach to investigate the adaptation of the alpine plant *Potentilla saundersiana* to the altitude gradient. Our proteomics results revealed a series of differential protein spots among the plant samples distributed along the altitude gradient (Figure 8), which were primarily involved in material and energy metabolism, antioxidant enzyme activities, defense response, protein degradation, cell wall synthesis, and hormone signal transduction. Further physiological analysis demonstrated the important role of primary metabolites, such as sugar and proline, in adaptation to environmental stress. Antioxidant enzyme activities against ROS damage also contribute to alpine plant adaptation to environmental stress. Protein accumulation during cell wall synthesis was involved in alpine plant tolerance to environmental stress by modulating primary and secondary cell wall structure. Further, epigenetic modifications of DNA stability, modulation of protein homeostasis through proteasome pathways, and the process of autophagy also affect plant adaptation to environmental stress. Adjustments of root architecture and leaf phenotype by hormone synthesis and signal transduction, particularly the dynamic competition of auxin and strictosidine, are also important for plant adaptation. In conclusion, we offer that *P. saundersiana* employs multiple strategies to enhance its adaptation to high-altitude environments in the Tibet plateau.

**Acknowledgements**

This work was supported by the Young Academic and Technical Leader Raising Foundation of Yunnan Province (No.2012HB041), the National Natural Sciences Foundation of China (No. 31170256 ), and the Major State Basic Research Development Program (2010CB951700).

References

1.	Xiong, L.; Schumaker, K. S.; Zhu, J.-K., Cell signaling during cold, drought, and salt stress. *The Plant Cell Online* **2002,** 14, (suppl 1), S165-S183.

2.	Chinnusamy, V.; Schumaker, K.; Zhu, J. K., Molecular genetic perspectives on cross‐talk and specificity in abiotic stress signalling in plants. *Journal of Experimental Botany* **2004,** 55, (395), 225-236.

3.	Körner, C., *Alpine Plant Life: Functional Plant Ecology of High Mountain Ecosystems; with 47 Tables*. Springer: 2003.

4.	Vinocur, B.; Altman, A., Recent advances in engineering plant tolerance to abiotic stress: achievements and limitations. *Current Opinion in Biotechnology* **2005,** 16, (2), 123-132.

5.	Gepstein, S.; Glick, B. R., Strategies to ameliorate abiotic stress-induced plant senescence. *Plant molecular biology* **2013**, 1-11.

6.	Foyer, C. H.; Noctor, G., Redox homeostasis and antioxidant signaling: a metabolic interface between stress perception and physiological responses. *The Plant Cell Online* **2005,** 17, (7), 1866-1875.

7.	Bowler, C.; Fluhr, R., The role of calcium and activated oxygens as signals for controlling cross-tolerance. *Trends in plant science* **2000,** 5, (6), 241-246.

8.	Serino, G.; Xie, Q., The Ever Expanding Role of Ubiquitin and SUMO in Plant Biology. *Journal of integrative plant biology* **2013,** 55, (1), 5-6.

9.	Guo, L.; Nezames, C. D.; Sheng, L.; Deng, X.; Wei, N., Cullin‐RING Ubiquitin Ligase Family in Plant Abiotic Stress PathwaysF. *Journal of integrative plant biology* **2013,** 55, (1), 21-30.

10.	Boyko, A.; Kovalchuk, I., Epigenetic Regulation of Genome Stability in Plants in Response to Stress. In *Epigenetic Memory and Control in Plants*, Springer: 2013; pp 41-56.

11.	Steffens, B.; Steffen-Heins, A.; Sauter, M., Reactive oxygen species mediate growth and death in submerged plants. *Frontiers in plant science* **2013,** 4.

12.	Dorji, T.; Totland, Ø.; Moe, S. R.; Hopping, K. A.; Pan, J.; Klein, J. A., Plant functional traits mediate reproductive phenology and success in response to experimental warming and snow addition in Tibet. *Global change biology* **2013,** 19, (2), 459-472.

13.	Westoby, M.; Reich, P. B.; Wright, I. J., Understanding ecological variation across species: area‐based vs mass‐based expression of leaf traits. *New Phytologist* **2013**.

14.	Poorter, H.; Niinemets, U.; Poorter, L.; Wright, I. J.; Villar, R., Causes and consequences of variation in leaf mass per area (LMA): a meta-analysis. *New Phytol* **2009,** 182, (3), 565-88.

15.	Zhou, J.; Lee, C.; Zhong, R.; Ye, Z.-H., MYB58 and MYB63 are transcriptional activators of the lignin biosynthetic pathway during secondary cell wall formation in Arabidopsis. *The Plant Cell Online* **2009,** 21, (1), 248-266.

16.	Sun, X.; Feng, Z.; Meng, L.; Zhu, J.; Geitmann, A., Arabidopsis ASL11/LBD15 is involved in shoot apical meristem development and regulates WUS expression. *Planta* **2013**, 1-12.

17.	Peng, Z.; Han, C.; Yuan, L.; Zhang, K.; Huang, H.; Ren, C., Brassinosteroid enhances jasmonate-induced anthocyanin accumulation in Arabidopsis seedlings. *J Integr Plant Biol* **2011,** 53, (8), 632-40.

18.	Zhao, M.-G.; Chen, L.; Zhang, L.-L.; Zhang, W.-H., Nitric reductase-dependent nitric oxide production is involved in cold acclimation and freezing tolerance in Arabidopsis. *Plant Physiology* **2009,** 151, (2), 755-767.

19.	Yang, L.; Tian, D.; Todd, C. D.; Luo, Y.; Hu, X., Comparative Proteome Analyses Reveal that Nitric Oxide Is an Important Signal Molecule in the Response of Rice to Aluminum Toxicity. *Journal of proteome research* **2013,** 12, (3), 1316-1330.

20.	Bai, X.; Yang, L.; Yang, Y.; Ahmad, P.; Yang, Y.; Hu, X., Deciphering the protective role of nitric oxide against salt stress at the physiological and proteomic levels in maize. *Journal of proteome research* **2011,** 10, (10), 4349-4364.

21.	He, Z.; Wang, Z.-Y.; Li, J.; Zhu, Q.; Lamb, C.; Ronald, P.; Chory, J., Perception of brassinosteroids by the extracellular domain of the receptor kinase BRI1. *Science Signaling* **2000,** 288, (5475), 2360.

22.	Edlund, A.; Eklof, S.; Sundberg, B.; Moritz, T.; Sandberg, G., A microscale technique for gas chromatography-mass spectrometry measurements of picogram amounts of indole-3-acetic acid in plant tissues. *Plant Physiology* **1995,** 108, (3), 1043-1047.

23.	Yoneyama, K.; Xie, X.; Sekimoto, H.; Takeuchi, Y.; Ogasawara, S.; Akiyama, K.; Hayashi, H.; Yoneyama, K., Strigolactones, host recognition signals for root parasitic plants and arbuscular mycorrhizal fungi, from Fabaceae plants. *New Phytologist* **2008,** 179, (2), 484-494.

24.	Pang, Q.; Chen, S.; Dai, S.; Chen, Y.; Wang, Y.; Yan, X., Comparative proteomics of salt tolerance in Arabidopsis thaliana and Thellungiella halophila. *Journal of proteome research* **2010,** 9, (5), 2584-2599.

25.	Wingler, A.; Purdy, S.; MacLean, J. A.; Pourtau, N., The role of sugars in integrating environmental signals during the regulation of leaf senescence. *Journal of Experimental Botany* **2006,** 57, (2), 391-399.

26.	Garg, A. K.; Kim, J.-K.; Owens, T. G.; Ranwala, A. P.; Do Choi, Y.; Kochian, L. V.; Wu, R. J., Trehalose accumulation in rice plants confers high tolerance levels to different abiotic stresses. *Proceedings of the National Academy of Sciences* **2002,** 99, (25), 15898-15903.

27.	Alcázar, R.; Altabella, T.; Marco, F.; Bortolotti, C.; Reymond, M.; Koncz, C.; Carrasco, P.; Tiburcio, A. F., Polyamines: molecules with regulatory functions in plant abiotic stress tolerance. *Planta* **2010,** 231, (6), 1237-1249.

28.	Knight, H.; Knight, M. R., Abiotic stress signalling pathways: specificity and cross-talk. *Trends in plant science* **2001,** 6, (6), 262-267.

29.	Besson-Bard, A.; Pugin, A.; Wendehenne, D., New insights into nitric oxide signaling in plants. *Annu. Rev. Plant Biol.* **2008,** 59, 21-39.

30.	Dixon, R.; Harrison, M.; Lamb, C., Early events in the activation of plant defense responses. *Annual review of phytopathology* **1994,** 32, (1), 479-501.

31.	Bai, X. G.; Chen, J. H.; Kong, X. X.; Todd, C. D.; Yang, Y. P.; Hu, X. Y.; Li, D. Z., Carbon monoxide enhances the chilling tolerance of recalcitrant Baccaurea ramiflora seeds via nitric oxide-mediated glutathione homeostasis. *Free Radic Biol Med* **2012,** 53, (4), 710-20.

32.	Foyer, C. H.; Noctor, G., Ascorbate and glutathione: the heart of the redox hub. *Plant Physiology* **2011,** 155, (1), 2-18.

33.	Chalker‐Scott, L., Environmental significance of anthocyanins in plant stress responses. *Photochemistry and photobiology* **1999,** 70, (1), 1-9.

34.	Dixon, R. A.; Paiva, N. L., Stress-induced phenylpropanoid metabolism. *The Plant Cell* **1995,** 7, (7), 1085.

35.	Winkel-Shirley, B., Biosynthesis of flavonoids and effects of stress. *Current opinion in plant biology* **2002,** 5, (3), 218-223.

36.	Brett, C. T.; Hillman, J. R., *Biochemistry of plant cell walls*. CUP Archive: 1985; Vol. 70.

37.	Showalter, A. M., Structure and function of plant cell wall proteins. *The Plant Cell* **1993,** 5, (1), 9.

38.	Cosgrove, D. J., Loosening of plant cell walls by expansins. *Nature* **2000,** 407, (6802), 321-326.

39.	Ichimura, K.; Shinozaki, K.; Tena, G.; Sheen, J.; Henry, Y.; Champion, A.; Kreis, M.; Zhang, S.; Hirt, H.; Wilson, C., Mitogen-activated protein kinase cascades in plants: a new nomenclature. *Trends in plant science* **2002,** 7, (7), 301-308.

40.	Xiao, W.; Sheen, J.; Jang, J.-C., The role of hexokinase in plant sugar signal transduction and growth and development. *Plant molecular biology* **2000,** 44, (4), 451-461.

41.	Palma, J. M.; Sandalio, L. M.; Javier Corpas, F.; Romero-Puertas, M. C.; McCarthy, I.; del Río, L. A., Plant proteases, protein degradation, and oxidative stress: role of peroxisomes. *Plant Physiology and Biochemistry* **2002,** 40, (6), 521-530.

42.	Vierstra, R. D., Protein degradation in plants. *Annual review of plant biology* **1993,** 44, (1), 385-410.

43.	Miura, K.; Hasegawa, P. M., Sumoylation and other ubiquitin-like post-translational modifications in plants. *Trends in cell biology* **2010,** 20, (4), 223-232.

44.	Bassham, D. C., Plant autophagy—more than a starvation response. *Current opinion in plant biology* **2007,** 10, (6), 587-593.

45.	Xiong, Y.; Contento, A. L.; Nguyen, P. Q.; Bassham, D. C., Degradation of oxidized proteins by autophagy during oxidative stress in Arabidopsis. *Plant Physiology* **2007,** 143, (1), 291-299.

46.	Chinnusamy, V.; Zhu, J.-K., Epigenetic regulation of stress responses in plants. *Current opinion in plant biology* **2009,** 12, (2), 133-139.

47.	Boyko, A.; Kovalchuk, I., Epigenetic control of plant stress response. *Environmental and molecular mutagenesis* **2008,** 49, (1), 61-72.

48.	Saez, A.; Rodrigues, A.; Santiago, J.; Rubio, S.; Rodriguez, P. L., HAB1–SWI3B interaction reveals a link between abscisic acid signaling and putative SWI/SNF chromatin-remodeling complexes in Arabidopsis. *The Plant Cell Online* **2008,** 20, (11), 2972-2988.

49.	Bari, R.; Jones, J. D., Role of plant hormones in plant defence responses. *Plant molecular biology* **2009,** 69, (4), 473-488.

50.	Rasmussen, A.; Mason, M. G.; De Cuyper, C.; Brewer, P. B.; Herold, S.; Agusti, J.; Geelen, D.; Greb, T.; Goormachtig, S.; Beeckman, T., Strigolactones suppress adventitious rooting in Arabidopsis and pea. *Plant Physiology* **2012,** 158, (4), 1976-1987.

**Figure Legends**

Figure 1: Effects of the altitude gradient on the phenotype of the whole plant (A), the root architecture (B), the leaf shape (C) and the stomatal size (D) in alpine *P. saundersiana*. The samples of *P. saundersiana* were collected at different altitudes sites (4350 m, 4600 m, 4800 m, 5100 m, and 5200 m) for further analysis. The whole plant phenotype (A, bar=15cm), the root architecture (B, bar= 3cm), and the leaf shape (C, bar=1.5cm) are shown with photographs; the stomatal shape was observed with scanning electron microscopy (SEM) analysis (D, bar=10 μm). At least 50 plant samples, or at least 200 leaves, were collected for analysis from each site.

Figure 2: Effects of the altitude gradient on the stomatal length, leaf nitrogen index, special leaf index and photosynthetic Fv/Fm ratio in *P. saundersiana.* The plant samples were collected at different altitudes (4300 m, 4600 m, 4800 m, 5100 m, and 5200 m) and the stomatal length (n=20 for each site), leaf nitrogen index (n=50 for each site), special leaf index (n=50 for each site) and photosynthetic Fv/Fm ratio (n=10 for each site) were measured. The data represent the means of three replicated experiments (±SE). The star symbol indicates a significant difference according to the t-test (p<0.05).

Figure 3: Dynamic protein changes in *P. saundersiana* at different altitudes. The plant samples were collected at different altitudes (4300 m, 4600 m, 4800 m, 5100 m, and 5200 m) and 1 mg of total protein was extracted and loaded into gels. (A) BR-20 stained 2-D gel of total protein. (B) Enlarged windows from panel A of the spot changes in the representative gels from samples at different altitudes.

Figure 4: Functional classification (A), Venn diagram analysis (B), and hierarchical clustering (C) for the expression protein profiles in *P. saundersiana* samples from different altitudes. The hierarchical cluster analysis was conducted using the cluster 3.0 and Treeview software (<http://bonsai.ims.u-tokyo.ac.jp/~mdehoon/software/cluster>). The different colors correspond to the log-transformed values of the protein change-fold ratio shown in the bar at the bottom of the figure. The enlarged figures show a set of identified expression proteins.

Figure 5: Changes in antioxidant enzymes and stress-related metabolites in *P. saundersiana* samples from different altitudes. (A) The effects of altitude on proline and sugar contents in *P. saundersiana* samples. (B) The effects of altitude on anthocyanin content in *P. saundersiana* samples. (C) The effects of altitude on H_2_O_2_ and O_2_^-^ contents in *P. saundersiana* samples. (D) The effects of altitude on antioxidant APX and SOD enzyme activities in *P. saundersiana* samples (Left, the quantified antioxidant enzyme activities; Right, in-gel staining activities). *P. saundersiana* samples were collected from different altitudes (4300 m, 4600 m, 4800 m, 5100 m, and 5200 m); the proline, sugar, anthocyanin and ROS contents and the antioxidant enzyme activities were determined. Data represent the means of five replicated experiments (±SE). The means denoted by different letters indicate significant differences according Tukey’s test (p<0.05).

Figure 6: Effects of the altitude gradient on cell wall structure in *P. saundersiana* samples from different altitudes. (A) The *P. saundersiana* samples were collected from different altitudes (4300 m, 4600 m, 4800 m, 5100 m, and 5200 m), and the cell wall structure was observed by transmission electron microscope (TEM). Upper: the cell wall structure by TEM; bottom: the enlarged figures from the windows in the upper section. Bars=1 mm. (B) The cell wall lignin distribution was observed by the special fluorescence dye calcofluor white. Bars=400 μm. Abbreviations: co, cortex; ep, epidermis; if, interfascicular fiber; xy, xylem.

Figure 7: Effects of the altitude gradient on differential expression protein accumulations and phytohormones. (A) Western blotting showing the effects of the altitude gradient on HSP, dehydrin, SOD, APX and Atg8a protein accumulations. (B) Effects of altitude gradient on auxin and strictosidine. *P. saundersiana* samples were collected from different altitudes (4300 m, 4600 m, 4800 m, 5100 m, and 5200 m), and the HSP, dehydrin, SOD, APX and Atg8a protein accumulations and auxin and strictosidine phytohormone contents were determined. Data represent the means of five replicated experiments (±SE). Means denoted by different letters indicate significant differences according Tukey’s test (p<0.05).

Figure 8: Schematic illustration of a proposed model for *P. saundersiana* adaptation to alpine environmental stress along the altitude gradient. The alpine plant *P. saundersiana* applied multiple strategies to enhance its adaptation to environmental stress along the slope with different altitudes (4350 m, 4600 m, 4800 m, 5100 m, and 5200 m). At the physiological level, the different samples show different SPA, root architectures, photosynthesis capabilities, stomatal sizes and cell wall structures. At the biochemical level, they synthesize different primary (such as proline and sugar) and secondary metabolites (such as flavones) and control hormone synthesis and signal transduction. A series of downstream defense and antioxidant enzyme activities also contribute to the plant’s adaptation to alpine environmental stress.

Table1: MS/MS identification of differentially expressed proteins in *P. saundersiana* at different altitudes

**Supplemental Information**

Supplemental Figure 1: The changes in soil temperature and rainfall along the slope at different altitudes.

Supplement Figure 2: PCA analyses of the contribution of different environmental factors to environmental stress response. (A) The contributing percentage of different components in our experiments. (B) The contribution of different environment factors in component 1 in our experiments.

Supplement Figure 3: The 2D figures of samples from altitudes of 4350 m (A), 4600 m (B), 4800 m (C), 5100 m (D), and 5200 m (E).

Tabel1 Indentification of proteins in *P. saundersiana* that are differentially expressed greater than 1.5-fold among different latitude gradient of location using MALDI-MS/MS analysis.

**Graphical Abstract**

This study investigated the alpine plant *Potentilla saundersiana* along the altitude gradient in the Northwestern Tibet Plateau. Our proteomics and physiological data demonstrate that *P. saundersiana* uses multiple strategies to adapt to alpine environmental stress, including 1) modulating root architecture, leaf phenotype, photosynthesis capability and cell wall structure; 2) regulating primary and secondary metabolites (such as sugar, proline and flavones) and plant hormone synthesis; 3) activating downstream defense response and antioxidant enzyme activities; 4) initiating novel proteasome or autophagy-mediated protein degradation mechanisms; and 5) controlling DNA stability at the epigenetic level. Therefore, we conclude that the alpine plant *P. saundersiana* evolved multiple strategies to adapt to environmental stress at high altitudes.

Tabel1 Indentification of proteins in *P. saundersiana* that are differentially expressed greater than 1.5-fold among different latitude gradient of location using MALDI-MS/MS analysis.

| Spot  No. | NCBI Accession No.^a^ | | Protein Name | | Species | Exp.  Mw/pI ^b^ | Theo. Mw/pI ^c^ | Score^d^ | Sc.^e^ | p Value^f^ | Ratio ^g^ | | | |  | | |
| --- | --- | --- | --- | --- | --- | --- | --- | --- | --- | --- | --- | --- | --- | --- | --- | --- | --- |
|  |  |  |  |  |  |  |  |  |  |  | 4600m  /4350m | 4800m  /4350m | 5100m  /4350m | 5200m  /4350m |  | | |
| Material and metabolism | | | | | | | | | | | | | | |  | | |
| 2 | gi\|1041248 | | pyrroline-5-carboxylate synthase | | *A.thaliana* | 77.70/5.89 | 62.4/5.83 | 168 | 8.5 | 0.03 | 1.11 | 1.42 | 1.89 | 2.11 |  | | |
| 7 | gi\|158512933 | | Soluble starch synthase 1, chloroplastic/amyloplastic | | *O．sativa* | 59.8/5.56 | 59.2/5.56 | 122 | 8.4 | 0.01 | 1.85 | 12.47 | 101.7 | 67.43 |  | | |
| 9 | gi\|3287832 | | (+)-delta-cadinene synthase | | *G.hirsutum* | 63.8/5.32 | 64.0/5.31 | 154 | 7.6 | 0.02 | 1.79 | 5.26 | 6.379 | 7.16 |  | | |
| 11 | gi\|300680834 | | Fructan 1-exohydrolase | | *L.chinensis* | 63.2/5.10 | 64.1/5.09 | 106 | 8.8 | 0.02 | 1.35 | 4.47 | 4.64 | 5.57 |  | | |
| 14 | gi\|75288633 | | Beta-glucosidase 22 | | *O. sativa* | 59.8/4.91 | 59.5/ 4.96 | 167 | 8.6 | 0.03 | -1.11 | -1.81 | -2.66 | -3.28 |  | | |
| 15 | gi\|190358253 | | ATP synthase subunit beta, chloroplastic | | *A.evecta* | 58.6/4.96 | 52.8/5.01 | 152 | 11.2 | 0.01 | -1.24 | 2.15 | -1.47 | 1.77 |  | | |
| 17 | gi\|334724538 | | Phospholipase A1-II 2 | | *O. sativa* | 49/5.56 | 45.1/5.69 | 89 | 8.9 | 0.02 | 1.13 | 1.49 | -7.52 | -13.51 |  | | |
| 18 | gi\|129916 | | Phosphoglycerate kinase, cytosolic | | *T.aestivum* | 44.2/5.69 | 42.1/5.64 | 95 | 11 | 0.02 | -1.29 | -2.89 | -3.07 | -5.67 |  | | |
| 20 | gi\|113683 | | Alpha-amylase isozyme 3E | | *O. sativa* | 46.2/5.19 | 46.1/5.14 | 99 | 10.3 | 0.03 | -1.25 | -1.55 | -2.02 | -2.21 |  | | |
| 23 | gi\|75279074 | | UDP-glycosyltransferase 71C1 | | *A.thaliana* | 56.2/4.78 | 53.8/4.83 | 103 | 17.7 | 0.01 | 1.42 | -4.12 | 2.74 | 3.25 |  | | |
| 24 | gi\|75161468 | | Glucan endo-1,3-beta-glucosidase 12 | | *A.thaliana* | 51/4.92 | 51.9/4.92 | 89 | 10.1 | 0.03 | -1.33 | 1.26 | -2.46 | -31.57 |  | | |
| 25 | gi\|12643259 | | Ribulose bisphosphate carboxylase/oxygenase activase | | *A.thaliana* | 48.4/5.06 | 46.2/5.09 | 115 | 16.7 | 0.01 | 1.67 | -1.54 | -1.32 | -3.35 |  | | |
| 35 | gi\|1345776 | | Acidic endochitinase WIN6.2B | | *P.trichocarpa* | 33/4.1 | 29.9/4.18 | 142 | 22.1 | 0.01 | 1.16 | -9.25 | -2.47 | -1.49 |  | | |
| 39 | gi\|121279 | | Glycinin A4 subunit | | *G. max* | 30.8/4.75 | 29.9/4.70 | 167 | 20.6 | 0.02 | 1.43 | 2.98 | 4.87 | 5.06 |  | | |
| 40 | gi\|12644069 | | Spermidine synthase 2 | | *A. thaliana* | 35.8/4.65 | 37.1/4.73 | 117 | 30 | 0.03 | 1.21 | 1.72 | 3.54 | 4.18 |  | | |
| 41 | gi\|3913641 | | Fructose-1,6-bisphosphatase, chloroplastic | | *O. sativa* | 36/4.76 | 38.8/4.68 | 154 | 23.7 | 0.02 | -1.13 | -1.86 | -7.99 | -1.28 |  | | |
| 47 | gi\|62900689 | | Probable plastid-lipid-associated protein 3, chloroplastic | | *O. sativa* | 38.4/4.14 | 35.1/4.13 | 127 | 21.1 | 0.01 | -1.56 | 1.1 | 2.94 | 3.19 |  | | |
| 49 | gi\|152013348 | | protein arginine N- methyltransferase | | *O. sativa* | 41.2/5/22 | 43.0/5.26 | 166 | 20.9 | 0.03 | 1.06 | 1.4 | 1.48 | 1.95 |  | | |
| 50 | gi\|93213522 | | trehalose-phosphate synthase | | *R. opacus* | 55.3/6.69 | 52.30/6.65 | 158 |  | 0.01 | 1.19 | 1.5 | 1.94 | 2.65 |  | | |
| 51 | gi\|75135272 | | Flavone 3'-O-methyltransferase 1 | | *O. sativa* | 39.6/5.4 | 39.6/5.41 | 216 | 27.2 | 0.01 | 2.14 | 2.67 | 5.46 | 6.99 |  | | |
| 54 | gi\|223635320 | | S-adenosylmethionine synthase | | *O. sativa* | 41.6/5.46 | 42.9/5.48 | 211 | 19.2 | 0.03 | -1.14 | -1.18 | -2.55 | -2.31 |  | | |
| 57 | gi\|121332 | | Glutamine synthetase cytosolic isozyme 1- 2 | | *O. sativa* | 37.8/5.75 | 39.2/5.73 | 169 | 25.5 | 0.02 | -1.17 | -1.22 | 2.23 | 2.15 |  | | |
| 58 | gi\|11131561 | | Cysteine synthase | | *B. juncea* | 35.6/5.76 | 33.9/5.67 | 176 | 29.2 | 0.04 | -1.01 | 1.28 | 1.33 | 1.57 |  | | |
| 60 | gi\|75213205 | | Endonuclease 1 | | *A.thaliana* | 33/5.85 | 31.9/5.83 | 155 | 32.8 | 0.01 | 1.27 | 1.85 | -3.66 | 3.25 |  | | |
| 62 | gi\|85690840 | | Tocopherol O-methyltransferase, chloroplastic | | *A.thaliana* | 32.8/5.75 | 32.9/5.80 | 113 | 13.0 | 0.04 | 1.03 | 2.53 | 1.95 | 2.91 |  | | |
| 63 | gi\|416674 | | ATP synthase subunit a | | *V. faba* | 31.8/5.56 | 32.5/5.56 | 95 | 8.3 | 0.02 | 2.49 | 13.11 | 13.41 | 21.93 |  | | |
| 65 | gi\|1708427 | | Isoflavone reductase | | *P.sativum* | 34.2/5.38 | 35.4/5.38 | 118 |  | 0.03 | 1.56 | 1.73 | 2.44 | 2.49 |  | | |
| 66 | gi\|3914503 | | Aspartate carbamoyltransferase 2, chloroplastic | | *P. sativum* | 36/5.42 | 36.4/5.47 | 95 | 27.0 | 0.02 | 1.15 | 1.19 | 1.52 | 1.75 |  | | |
| 74 | gi\|158515403 | | Vegetative storage protein 1 | | *A.thaliana* | 27.6/5.32 | 28.5/5.34 | 84 | 23.3 | 0.03 | 2.78 | 5.02 | 12.7 | 9.11 |  | | |
| 79 | gi\|75119480 | | Heme oxygenase 1, chloroplastic | | *O. sativa* | 25.8/5.12 | 25.6/5.22 | 79 | 27.3 | 0.01 | 1.38 | 4.47 | 4.14 | 4.77 |  | | |
| 84 | gi\|73921672 | | Allene oxide cyclase 2, chloroplastic | | *A.thaliana* | 16.8/5.05 | 19.4/5.39 | 114 | 36.4 | 0.03 | -1.08 | 1.29 | 2.45 | 2.77 |  | | |
| 85 | gi\|1171937 | | Oxalate oxidase 2 | | *H.vulgare* | 22.2/5.02 | 21.5/5.78 | 143 | 21.9 | 0.03 | -1.25 | -1.55 | -3.15 | -3.24 |  | | |
| 97 | gi\|391359267 | | Endonuclease 5 | | *A.thaliana* | 32.6/6.38 | 31.4/6.44 | 110 | 34.8 | 0.03 | -1.15 | 2.62 | 2.62 | 6.33 |  | | |
| 99 | gi\|134918 | | 12S seed storage globulin 1 acidic chain | | *A. sativa* | 32.6/6.58 | 33.2/6.66 | 92 | 17.6 | 0.01 | 1.46 | 2.28 | 3.22 | 4.56 |  | | |
| 100 | gi\|544011 | | Basic 30 kDa endochitinase | | *S.lycopersicon* | 31.8/6.64 | 31.1/6.57 | 96 | 23.9 | 0.03 | 5.36 | 22.14 | 26.82 | 35.42 |  | | |
| 101 | gi\|11133373 | | Malate dehydrogenase, cytoplasmic | | *M. sativa* | 36/6.42 | 35.5/6.40 | 145 | 37.1 | 0.02 | 1.45 | 2.14 | 3.88 | 4.87 |  | | |
| 102 | gi\|75151303 | | Glutamine synthetase cytosolic isozyme 1- 5 | | *A.thaliana* | 37.6/6.26 | 38.9/6.19 | 126 | 28.6 | 0.04 | -1.03 | -1.78 | -2.04 | -2.66 |  | | |
| 103 | gi\|1168493 | | Arginase | | *A.thaliana* | 35.6/6.11 | 37.3/6.11 | 184 | 25.7 | 0.03 | 1.09 | 6.71 | 10.55 | 10.56 |  | | |
| 105 | gi\|462139 | | Glyceraldehyde-3-phosphate dehydrogenase, cytosolic | | *P.subspatens* | 37.8/6.06 | 36.7/6.19 | 204 | 25.2 | 0.03 | 1.81 | 2.75 | 4.14 | 5.18 |  | | |
| 108 | gi\|78099755 | | Alpha-amylase isozyme 3D | | *O. sativa* | 45/5.77 | 45.4/5.75 | 221 | 21.1 | 0.01 | -1.74 | 1.41 | 1.31 | 1.61 |  | | |
| 111 | gi\|75303413 | | Ornithine decarboxylase | | *C.annuum* | 46.2/5.90 | 46.9/5.80 | 168 | 19.5 | 0.04 | 1.19 | 1.69 | 1.89 | 2.11 |  | | |
| 112 | gi\|231802 | | Chalcone synthase D | | *P. hybrida* | 46/5.92 | 45.9/5.97 | 164 | 23.2 | 0.02 | 1.06 | 1.06 | 1.73 | 2.26 |  | | |
| 115 | gi\|12229807 | | Glutamate dehydrogenase 1 | | *A.thaliana* | 46.2/5.92 | 44.5/6.38 | 113 | 26.5 | 0.03 | -6.62 | -5.53 | -5.03 | -2.88 |  | | |
| 117 | gi\|129940 | | Exopolygalacturonase | | *Z. mays* | 42.6/6.62 | 41.0/6.60 | 96 | 23.5 | 0.03 | 2.05 | 6.3 | 6.12 | 8.94 |  | | |
| Antioxidant enzyme and redox enzyme | | | | |  | | | | | | | | | |  | | |
| 42 | gi\|150421613 | | Peroxidase 36 | | *A.thaliana* | 36/4.82 | 35.2/4.78 | 99 | 21.2 | 0.03 | 1.48 | 2.48 | 8.38 | 8.91 |  | | |
| 71 | gi\|88909669 | | Probable L-ascorbate peroxidase 5, chloroplastic | | *O. sativa* | 30.8/5.2 | 30.3/5.23 | 84 | 31.3 | 0.01 | 1.03 | 1.29 | 5.34 | 5.75 |  | | |
| 75 | gi\|134646 | | Superoxide dismutase [Fe], chloroplastic | | *G. max* | 25/5.32 | 25.3/5.42 | 116 | 36.3 | 0.03 | 1.22 | 1.82 | 5.15 | 5.37 |  | | |
| 76 | gi\|353558646 | | Glutathione S-transferase U21 | | *A.thaliana* | 24.6/5.42 | 25.6/5.42 | 152 | 39.2 | 0.04 | 1.57 | 2.13 | 6.19 | 6.82 |  | | |
| 88 | gi\|728873 | | L-ascorbate peroxidase 1, cytosolic | | *A.thaliana* | 27.8/5.71 | 27.4/5.72 | 220 | 36.4 | 0.02 | 5.47 | 21.27 | 39.01 | 42.4 |  | | |
| 91 | gi\|1174391 | | Superoxide dismutase [Mn] 3.4, mitochondrial | | *Z. mays* | 22.4/5.88 | 22.4/5.89 | 129 | 36.1 | 0.03 | 1.48 | 7.94 | 13.67 | 13.78 |  | | |
| 93 | gi\|75324900 | | 1-Cys peroxiredoxin PER1 | | *T.aestivum* | 24.2/6.08 | 23.9/6.08 | 152 | 40.4 | 0.01 | 1.42 | 1.82 | 4.89 | 6.71 |  | | |
| 96 | gi\|5902677 | | Thioredoxin domain-containing protein 9 homolog | | *A.thaliana* | 24.2/6.35 | 24.4/6.35 | 106 | 33.2 | 0.01 | -1.01 | 1.4 | 2.93 | 3.98 |  | | |
| 107 | gi\|21542422 | | Cystathionine gamma-synthase, chloroplastic | | *A.thaliana* | 40.6/5.78 | 41.7/5.80 | 177 | 19.2 | 0.01 | 1.01 | 1.09 | 1.25 | 1.95 |  | | |
| 109 | gi\|50400716 | | Monodehydroascorbate reductase | | *P. sativum* | 46/5.76 | 47.3/5.80 | 157 | 24.9 | 0.01 | 1.12 | 1.15 | 1.25 | 1.82 |  | | |
| DNA impaire and Epigeneitcs | | | | | | | | | | | | | | |  | | |
| 21 | gi\|75331380 | | SWI/SNF complex subunit SWI3A | | *A.thaliana* | 63.8/4.76 | 57.5/4.80 | 184 | 9.2 | 0.04 | 1.55 | 2.07 | 2.01 | 2.42 |  | | |
| 32 | gi\|55976306 | | Probable DNA repair protein RAD23 | | *O. sativa* | 45/4.21 | 41.7/4.34 | 138 | 19.9 | 0.02 | -1.26 | 2.91 | 3.42 | 5.44 |  | | |
| Horomone related | | | | |  | | | | | | | | | |  | | |
| 1 | gi\|75261153 | | Auxin response factor 8 | | *O. sativa* | 74.5/6.59 | 73.8/6.57 | 213 | 6.0 | 0.03 | -1.02 | -1.16 | 1.46 | 1.67 |  | | |
| 3 | gi\|62900128 | | Indole-3-acetic acid-amido synthetase GH3.5 | | *A. thaliana* | 63.4/5.75 | 69.2/5.72 | 185 | 7.8 | 0.01 | -7.22 | 1.26 | 1.38 | 2.02 |  | | |
| 6 | gi\|37078118 | | ETHYLENE INSENSITIVE 3-like 2 protein | | *A.thaliana* | 58.2/5.70 | 59.2/5.75 | 204 | 11.8 | 0.03 | 1.24 | 1.81 | 2.48 | 1.97 |  | | |
| 10 | gi\|75104297 | | DELLA protein RGA2 | | *B. juncea* | 64/5.28 | 63.3/5.28 | 89 | 7.6 | 0.02 | 1.49 | 11.66 | 14.96 | 17.58 |  | | |
| 37 | gi\|75219968 | | Ethylene-responsive transcription factor ERF060 | | *A.thaliana* | 32.2/4.9 | 30.9/4.86 | 84 | 34.6 | 0.01 | 1.27 | 1.46 | 2.37 | 3.26 |  | | |
| 45 | gi\|148886766 | | 1-aminocyclopropane-1-carboxylate oxidase 1 | | *O. sativa* | 40/4.95 | 36.3/5.03 | 105 | 36.7 | 0.03 | 1.37 | 1.74 | 3.73 | 4.48 |  | | |
| 56 | gi\|75243635 | | IAA-amino acid hydrolase ILR1-like 4 | | *O. sativa* | 42.8/5.72 | 43.6/5.75 | 116 | 17.9 | 0.03 | 2.05 | 4.59 | 6.64 | 5.79 |  | | |
| 67 | gi\|398994 | | 1-aminocyclopropane-1-carboxylate oxidase | | *A.deliciosa* | 36.2/5.3 | 36.2/5.34 | 89 | 37.3 | 0.01 | -1.34 | 1.09 | 2.48 | 2.67 |  | | |
| 69 | gi\|267045 | | Strictosidine synthase | | *C.roseus* | 34.8/5.2 | 35.7/5.12 | 135 | 24.4 | 0.03 | 1.12 | -0.34 | -1.23 | -2.48 |  | | |
| 77 | gi\|17365900 | | Auxin-responsive protein IAA19 | | *A.thaliana* | 22.6/5.22 | 21.4/5.25 | 221 | 29.4 | 0.02 | -1.03 | 2.31 | 3.86 | 4.55 |  | | |
| 82 | gi\|1703042 | | ABA-responsive protein ABR17 | | *P.sativum* | 27.4/4.28 | 16.6/5.05 | 204 | 45.2 | 0.04 | 1.43 | 2.43 | 2.75 | 3.04 |  | | |
| 89 | gi\|11131315 | | IAA11_ARATH | | *A.thaliana* | 27.6/5.82 | 26.5/5.82 | 117 | 30.5 | 0.01 | 1.12 | 2.03 | 1.23 | 1.25 |  | | |
| 92 | gi\|75170450 | | Abscisic acid receptor PYL8 | | *A thaliana* | 20.8/6.08 | 21.4/6.07 | 168 | 41.0 | 0.04 | 1.72 | 4.47 | 5.47 | 7.01 |  | | |
| 104 | gi\|75336145 | | Gibberellin receptor GID1A | | *A.thaliana* | 37.6/6.11 | 38.6/6.16 | 153 | 22.3 | 0.04 | -2.07 | 1.23 | 4.78 | 10.89 |  | | |
| Protein kinase and phosphatase | | | | | | | | | | | | | | |  | | |
| 5 | | gi\|11386884 | Hexokinase-2 | *A.thaliana* | | 54.6/5.71 | 52.3/5.45 | 217 | 8.2 | 0.02 | -3.13 | 1.88 | 1.93 | 2.35 |  | | |
| 13 | | gi\|116054 | Calcium-dependent protein kinase SK5 | *G. max* | | 59.4/5.24 | 57.2/5.22 | 174 | 8.9 | 0.03 | -1.21 | -1.29 | -1.42 | -1.69 |  | | |
| 30 | | gi\|122212399 | protein phosphatase 2C | *O. sativa* | | 47.6/4.56 | 45.6/4.53 | 182 | 29.4 | 0.01 | 2.18 | 2.36 | 2.54 | 2.76 |  | | |
| 34 | | gi\|75324701 | CDK-activating kinase;es CDKB | *G. max* | | 38.8/4.06 | 35.1/4.09 | 190 | 18.9 | 0.03 | -1.35 | -3.07 | 0.32 | -3.36 |  | | |
| 44 | | gi\|71153749 | Serine/threonine-protein kinase SAPK10 | *O. sativa* | | 38.0/4.74 | 40.7/4.80 | 192 | 19.3 | 0.04 | 1.11 | -1.32 | 0.61 | -5.94 |  | | |
| 53 | | gi\|75321465 | Mitogen-activated protein kinase kinase 1 | *A.thaliana* | | 39.8/5.58 | 39.5/5.54 | 147 | 19.5 | 0.01 | -1.02 | -1.26 | -2.48 | -2.62 |  | | |
| 106 | | gi\|75333969 | Mitogen-activated protein kinase kinase 6 | *A.thaliana* | | 40/5.95 | 39.8/5.96 | 150 | 18.3 | 0.03 | 2.14 | 2.31 | 2.56 | 3.76 |  | | |
| 110 | | gi\|21431817 | Protein phosphatase 2C 56 | *A.thaliana* | | 47.2/5.75 | 47.5/5.80 | 95 | 24.4 | 0.02 | 1.09 | 1.45 | 1.6 | -5.42 |  | | |
| 114 | | gi\|75335204 | Mitogen-activated protein kinase 11 | *A.thaliana* | | 42.8/6.08 | 42.4/6.02 | 82 | 26.6 | 0.03 | 2.41 | 12.77 | 16.36 | 17.57 |  | | |
| 116 | | gi\|2499614 | Mitogen-activated protein kinase homolog NTF3 | *N .tabacum* | | 45/6.41 | 42.7/6.35 | 113 | 26.9 | 0.03 | 1.79 | 2.63 | 2.91 | 3.69 |  | | |
| Cell structure and division | | | | | | | | | | | | | | |  | | |
| 4 | gi\|85813772 | | Xylan-1,4-beta-xylosidase | *P. trichocarpa* | | 81.4/6.03 | 83.4/6.12 | 165 |  | 0.01 | -6.38 | 1.15 | -1.05 | 1.46 |  | | |
| 19 | gi\|5902735 | | Actin | *S.dubia* | | 43/5.21 | 41.8/5.22 | 135 | 8.7 | 0.01 | -1.09 | -1.4 | -2.28 | -3.28 |  | | |
| 22 | gi\|332645944 | | cellulose synthase | *A.thaliana* | | 61.4/6.30 | 60.4/5.11 | 128 |  | 0.01 | -1.56 | -9.98 | 2.74 | 3.03 |  | | |
| 29 | gi\|182657420 | | Actin-related protein 7 | *O. sativa* | | 42.2/4.76 | 39.6/4.73 | 188 | 21.1 | 0.03 | 1.55 | -1.19 | -1.91 | -1.39 |  | | |
| 33 | gi\|363548415 | | Cell division protein FtsZ | *H. mediterranei* | | 42.5/4.28 | 39.5/4.24 | 126 | 28.9 | 0.02 | 9.71 | 13.52 | 16.42 | 31.02 |  | | |
| 38 | gi\|115502169 | | Expansin-B12 | *O. sativa* | | 30.6/4.55 | 29.8/4.59 | 135 | 16.0 | 0.02 | 1.12 | 1.26 | 0.66 | -2.04 |  | | |
| 64 | gi\|75289696 | | Cyclin-D6-1 | *O. sativa* | | 32.6/5.52 | 34.2/5.50 | 192 | 25.9 | 0.02 | -2.78 | 1.81 | 3.77 | 5.53 |  | | |
| 78 | gi\|115502179 | | Expansin-A20 | *O. sativa* | | 24/5.14 | 23.6/4.14 | 96 | 20 | 0.04 | -1.4 | -3.3 | -14.69 | -2.27 |  | | |
| 90 | gi\|2960360 | | caffeoyl-CoA 3-O-methyltransferase | *P. trichocarpa* | | 27.9/5.33 | 24.6/5.93 | 195 |  | 0.04 | -1.55 | -2.02 | -1.83 | 1.03 |  | | |
| 94 | gi\|147636402 | | Cyclin-C1-1 | *A. thaliana* | | 28.6/6.06 | 29.9/6.00 | 104 | 31.2 | 0.01 | 1.73 | 2.74 | 4.25 | 6.65 |  | | |
| 98 | gi\|75252852 | | Expansin-like A4 | *O. sativa* | | 32.4/6.46 | 30.9/6.27 | 125 | 25.2 | 0.01 | 1.38 | 2.12 | 2.52 | 3.38 |  | | |
| 118 | gi\|75294998 | | Cyclin-A31 | *O. sativa* | | 39.6/6.78 | 41.3/6.85 | 182 |  | 0.03 | 2.42 | 4.85 | 8.65 | 10.02 |  | | |
| Defense response protein | | | | | | | | | | | | | | |  | | |
| 8 | gi\|22002057 | | Low-temperature-induced 65 kDa protein | *A.thaliana* | | 61.4/5.05 | 65.9/5.05 | 117 | 8.4 | 0.03 | 1.69 | 2.46 | 4.34 | 4.94 |  | | |
| 12 | gi\|75308864 | | Heat shock 70 kDa protein 18 | *A.thaliana* | | 62.8/5.2 | 68.4/5.22 | 145 | 7.5 | 0.03 | 1.36 | 4.11 | 6.41 | 7.74 |  | | |
| 61 | gi\|118926 | | Desiccation-related protein PCC13-6 | *C.plantagineum* | | 32.6/5.8 | 31.0/5.78 | 126 | 28.1 | 0.01 | -1.19 | -1.35 | -19.01 | -7.27 |  | | |
| 72 | gi\|1169018 | | Dehydrin COR410 | *T.aestivum* | | 28/5.18 | 28.2/5.20 | 117 | 23.3 | 0.02 | 1.17 | 1.28 | 3.58 | 3.94 |  | | |
| 80 | gi\|75291085 | | Chitinase 6 | *O.sativa* | | 26/4.76 | 26.5/4.57 | 137 | 25.1 | 0.01 | -1.23 | -1.01 | 1.1 | 1.28 |  | | |
| 83 | gi\|171769904 | | Dehydration-responsive element-binding protein 1C | *O. sativa* | | 22.2/4.4 | 23.1/5.01 | 192 | 29.4 | 0.04 | -1.46 | 3.75 | -5.05 | -5.42 |  | | |
| 86 | gi\|75279026 | | 17.7 kDa class I heat shock protein | *S.peruvianum* | | 18.6/5.4 | 17.6/5.42 | 115 | 40.3 | 0.03 | -1.63 | 2.79 | 2.85 | 3.85 |  | | |
| Protein degradation and refolding | | | | | | | | | | | | | | |  |  | Protein degradation and refolding |
| 27 | gi\|75135758 | | Autophagy protein 5a | *O. sativa* | | 42/4.96 | 42.3/5.01 | 104 | 22.1 | 0.04 | 1.13 | 1.19 | 1.86 | 1.14 |  | | |
| 36 | gi\|75301658 | | E3 ubiquitin-protein ligase RING1-like | *A. thaliana* | | 33.6/4.26 | 35.7/4.34 | 113 | 27.7 | 0.01 | 1.09 | 2.39 | 1.91 | 2.45 |  | | |
| 43 | gi\|158563947 | | E3 ubiquitin-protein ligase SINA-like 5 | *A.thaliana* | | 34.6/4.86 | 37.3/4.90 | 128 | 25.3 | 0.01 | 1.55 | -2.26 | 5.15 | 5.64 |  | | |
| 68 | gi\|384950747 | | SUMO-activating enzyme subunit 1B-1 | *A.thaliana* | | 34.6/5.15 | 35.6/5.14 | 116 | 36.9 | 0.03 | 1.11 | 1.33 | 1.82 | 2.55 |  | | |
| 81 | gi\|136644 | | Ubiquitin-conjugating enzyme E2-23 kDa | *T.aestivum* | | 27.2/4.55 | 21.1/4.40 | 152 | 28.8 | 0.02 | 1.04 | 1.21 | 1.33 | 1.47 |  | | |
| 113 | gi\|28558165 | | 26S protease regulatory subunit 7 | *O. sativa* | | 46/6.04 | 47.6/6.02 | 155 | 24.4 | 0.03 | 1.47 | 2.37 | 2.4 | 2.87 |  | | |
| Transcriptional factor | | | | | | | | | | | | | | |  | | |
| 16 | gi\|75298093 | | Heat stress transcription factor A-1 | *O. sativa* | | 57.6/5.04 | 55.2/4.97 | 197 | 9.5 | 0.04 | 1.48 | 1.97 | 3.89 | 5.69 |  | | |
| 26 | gi\|122247345 | | Heat stress transcription factor A-9 | *O. sativa* | | 43.6/5.09 | 45.4/5.03 | 192 | 13.9 | 0.03 | 1.09 | 1.49 | 2.27 | 2.38 |  | | |
| 28 | gi\|729774 | | Heat shock factor protein HSF30 | *S.peruvianum* | | 42.2/4.82 | 40.1/4.78 | 178 | 19.4 | 0.04 | 1.26 | 2.83 | 3.79 | 5.38 |  | | |
| 31 | gi\|148887454 | | Probable WRKY transcription factor 10 | *A.thaliana* | | 53.2/4.3 | 54.5/4.24 | 95 | 12.2 | 0.01 | -1.19 | -1.34 | -1.75 | -2.04 |  | | |
| 46 | gi\|75297913 | | Heat stress transcription factor A-2a | *O. sativa* | | 39.8/5.02 | 40.8/4.98 | 170 | 18.1 | 0.01 | -1.25 | 1.12 | 1.7 | 2.21 |  | | |
| 48 | gi\|75317981 | | Transcription factor MYB12 | *A.thaliana* | | 40.8/5.14 | 41.3/5.05 | 90 | 15.1 | 0.03 | 1.58 | 2.76 | 4.92 | 4.84 |  | | |
| 52 | gi\|75264295 | | Heat stress transcription factor A-9 | *A. thaliana* | | 37/5/58 | 38.1/5.49 | 152 | 32.6 | 0.04 | -1.07 | -1.27 | -1.46 | -1.54 |  | | |
| 55 | gi\|75337491 | | Transcription factor MYB28 | *Arthaliana* | | 42.6/5.72 | 41.1/5.71 | 168 | 15.3 | 0.02 | 1.23 | 2.21 | 3.35 | 6.2 |  | | |
| 59 | gi\|122771 | | Transcription factor HBP-1a | *T.aestivum* | | 35.8/5.91 | 32.8/6.01 | 103 | 27.5 | 0.01 | -1.34 | -3.31 | -3.83 | -4.21 |  | | |
| 70 | gi\|547958 | | Myb-related protein Hv33 | *H.vulgare* | | 34.2/5.25 | 33.0/5.17 | 127 | 25.2 | 0.01 | 1.76 | 2.48 | 2.6 | 6.16 |  | | |
| 73 | gi\|75277249 | | Heat stress transcription factor B-3 | *A.thaliana* | | 28.4/5.41 | 28.3/5.28 | 127 | 28.7 | 0.03 | 1.04 | 1.46 | 2.33 | 2.42 |  | | |
| 87 | gi\|84029365 | | Nuclear transcription factor Y subunit C- 2 | *A.thaliana* | | 22.2/5.68 | 23.1/5.66 | 94 | 34.2 | 0.03 | 1.18 | 2.23 | 10.44 | 14.37 |  | | |
| 95 | gi\|75171495 | | Transcription factor TCP6 | *A. thaliana* | | 27.6/6.2 | 27.6/6.11 | 122 | 25.9 | 0.02 | 1.99 | 2.15 | 2.18 | 3.25 |  | | |

a,Database accession numbers according to NCBInr

b, Experimental Mw/pI

c, Theoretical Mw/pI

d,The Mascot search score against the database of NCBInr

e, Sequences coverage

f, protein spots showed a significant change in abundance( fold change) by a factor>1.5-fold compared to the control analyzed by LSD test. A p-value of <0.05 was considered statistically significant

g, the different protein spots intensity ratios of samples at different latitude. 4600m/4350m: the different protein spot intensity ratio at 4600m to at4350m as the control; 4800m/4350m: the different protein spot intensity ratio at 4800m to at4350m as the control; 5100m/4350m: the different protein spot intensity ratio at 5100m to at4350m as the control; 5200m/4350m: the different protein spot intensity ratio at 5200m to at 4350m as the control.
